# Supplementary material for: Diversity of Conopeptides and Their Precursor Genes of Conus Litteratus
Source: Mar Drugs. 2020 Sep 14;18(9):464. doi: 10.3390/md18090464 (PMC7551347; doi:10.3390/md18090464)
Supplement: Supplementary file 1 [file marinedrugs-18-00464-s001.zip › Supplementary/S10-RNA preparation in each group and other supporting information.docx]

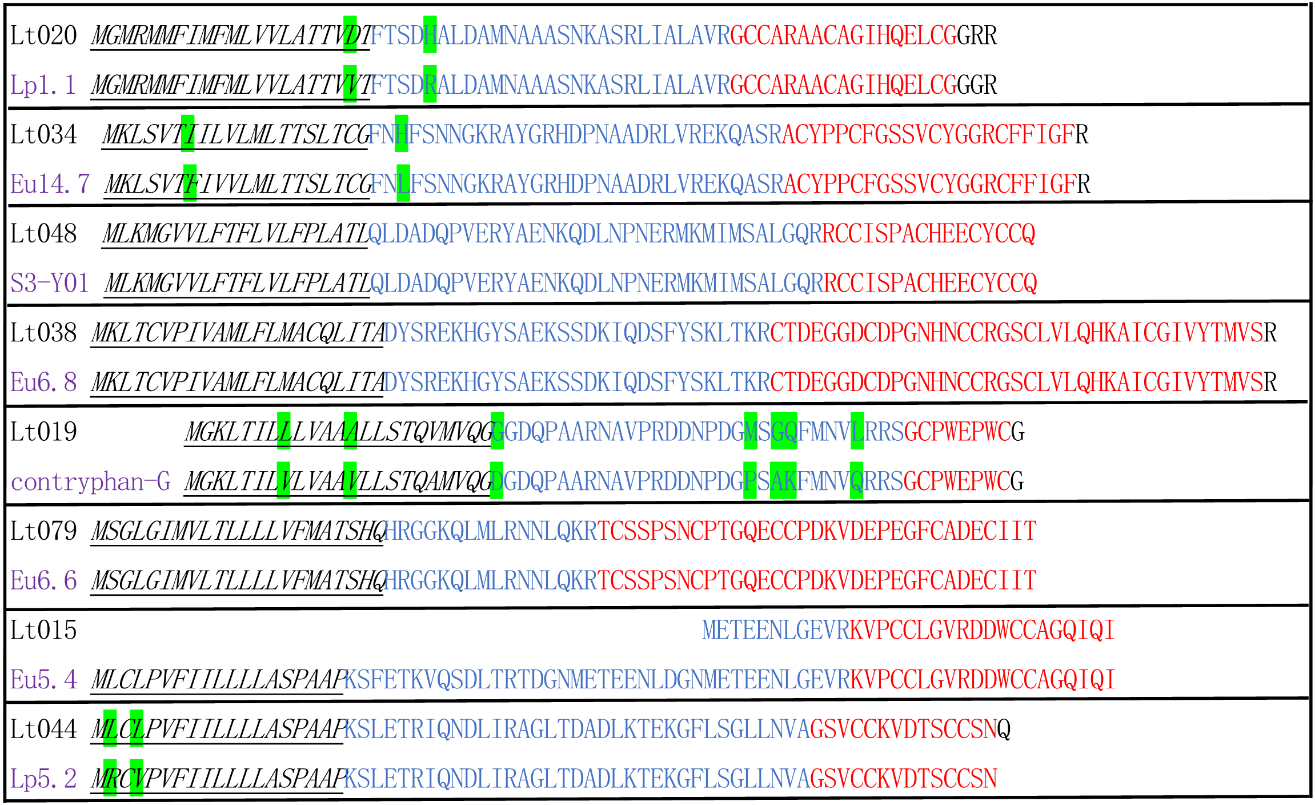


Figure S1. The alignment of highly homologous sequences in Table 2 reported from other *Conus* species.


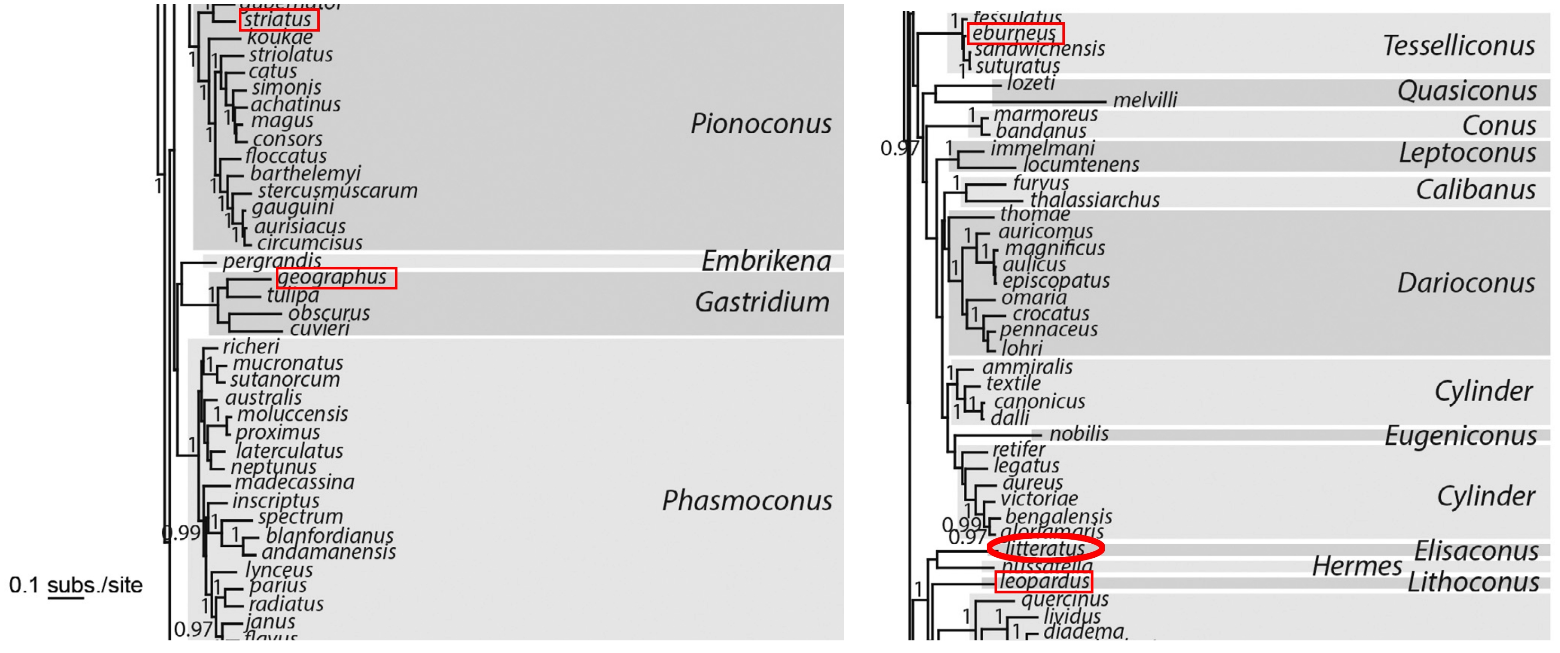


Figure S2. Bayesian tree based on a concatenation of the COI, 16S and 12S genes for the reduced dataset of 326 specimens cited from reference 5. *Conus* species with red frame may generate a few same conopeptide sequences compare to *C. literatus*.


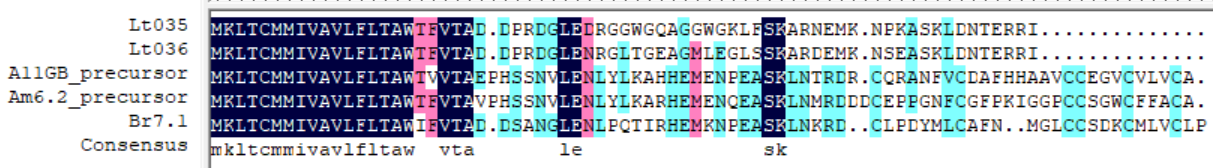


Figure S3. Alignment of O1-superfamily conotoxins with or without rich cysteines.


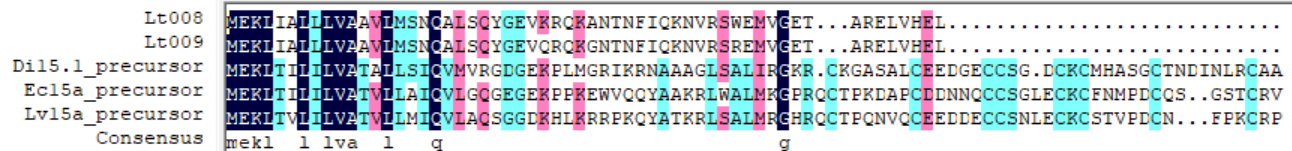


Figure S4. Alignment of O2-superfamily conotoxins with or without rich cysteines.


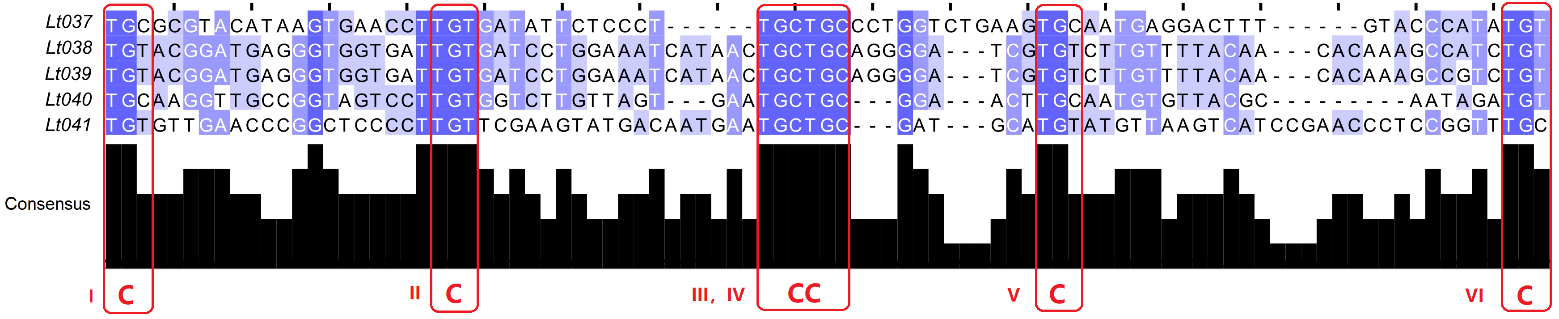


Figure S5. Codon usage for cysteine in the O1-superfamily conotoxin genes.

M 1 2 3 4 5 6 7 8 9 10 11 12 13 14 15 C

**
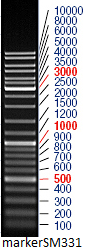

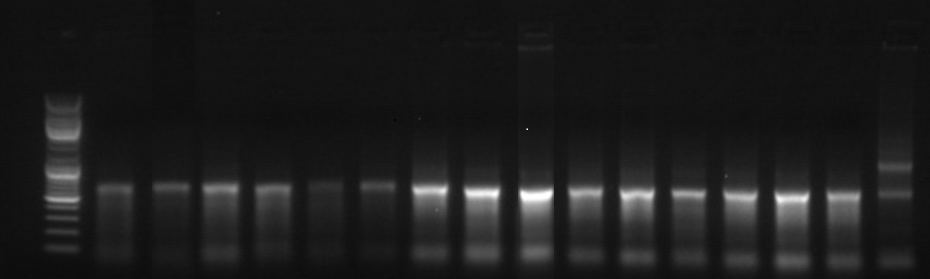
**

Figure S6. Total RNAs of each venom ducts were extracted from different individuals of *C. litteratus.* M: RNA Marker, 1-5: Small size cone snail; 6-10: Medium size cone snail; 11-15: Big size cone snail; C: Positive control.

Table S1. Total RNA concentrations for 15 specimens.

| No. | 1 | 2 | 3 | 4 | 5 | 6 | 7 | 8 | 9 | 10 | 11 | 12 | 13 | 14 | 15 |
| --- | --- | --- | --- | --- | --- | --- | --- | --- | --- | --- | --- | --- | --- | --- | --- |
| Conc.  (ng/μl) | 476 | 117 | 726 | 628 | 308 | 658 | 1160 | 1600 | 514 | 1120 | 1320 | 1080 | 1800 | 1140 | 1380 |

Table S2. Final RNA concentration of mixed samples of each group.

| Group | Conc. (ng/μl) |
| --- | --- |
| Small size cone snail (Figure S5, 1-5) | 258 |
| Medium size cone snail (Figure S5, 6-10) | 335 |
| Big size cone snail (Figure S5, 11-15) | 300 |


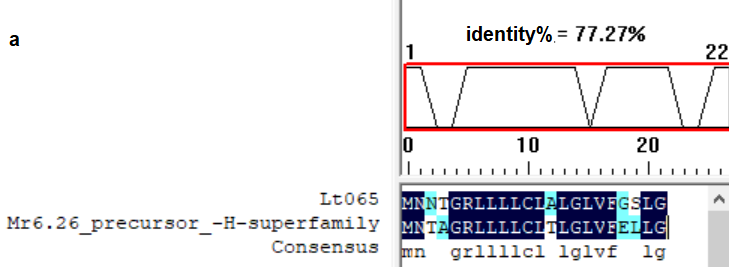

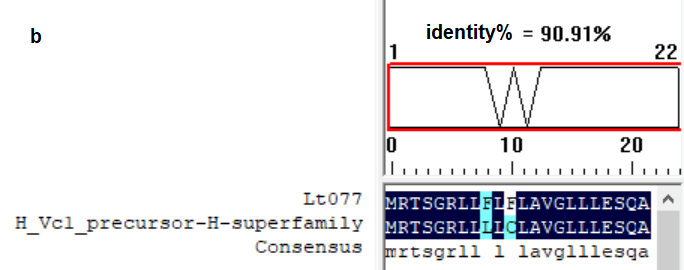


Figure S7. Alignment of the signal region of Lt065, Lt077 with the reported conotoxin belonging to H-superfamily in Conosever.


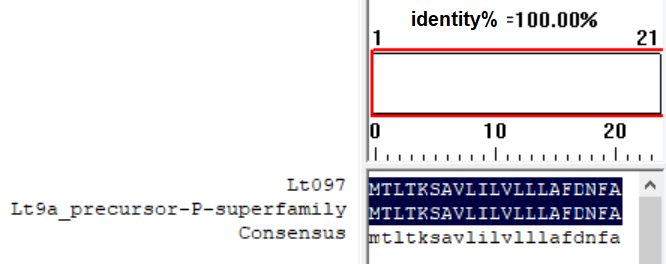


Figure S8. Alignment of the signal region of Lt097 with the reported conotoxin belonging to P-superfamily in Conosever.

Generally, when the identity between two signal sequences is >75%, it is designated as members of the same gene superfamily according to Conoserver classification rules. Signal region of Lt065 shared 77.27% identity with known Mr6.26 precursor. Signal region of Lt077 shared 90.91% identity with known H_Vc1 precursor. Both Mr6.26 and H_Vc1 precursors belong to H-superfamily members reported inConoserver. So, Lt065 and Lt077 should be included in H-superfamily (S10-Figure S6). Signal sequence of Lt097 shared 100% identity with known Lt9a precursor, which belongs to P-superfamily in Conoserver. Then Lt097 should be included in the P-superfamily too (S10-Figure S7).
