# Supplementary material for: Diversity of Conopeptides and Their Precursor Genes of Conus Litteratus
Source: Mar Drugs. 2020 Sep 14;18(9):464. doi: 10.3390/md18090464 (PMC7551347; doi:10.3390/md18090464)
Supplement: Supplementary file 1 [file marinedrugs-18-00464-s001.zip › Supplementary/S2-CDS of all conotoxin.docx]

>Lt001

ATGGCCATGAACATGTGGACGACGATCAGCCTGTTTGTAGTGGTTGTCGTGGCAGCCACTGTCGTTGGCTCCACTCCGTTACAAGAACAAGGCCTCAGCCTCGGTGAACGCCGTAACCCTGGATGCTGCGCCTGGGAATTCTATGACTGTCTTATACGAGGCAGGGTATGGAGGAGCCGTTTCTCTTTTTGCTACAGCATGGCCTCCAGCATTTGTACAAGAGGTCGTGCTGGTGGCTGCTGCCCTGGGTTGTTGCACTGTTTTGAAGTGTGCACGTCGAGAGATAGCGCAGTCTGCTATTACCGCTGCAAATTTGCGTCTTGCTGA

>Lt002

ATGGCGGTGCCAATGCTGGGAGGGTTTGGCGGCGGTCTCCGGGCTACTTTTTCAGGATGTTCCGTGTGCGGGTTTGCTGCCGCTGGAGATGCTGGCGAACCCGTGGCGGCTCCTTGTTTCTGCTGGTGGAGCTCATGGTTGAATGGGAGGTGGGTTCTGGTGAGGCGTAGTTCCAGCAGTTCTGGCACCTGTTCTGTAGTGAGTTGA

>Lt003

ATGGACGCACGTCGTGTAGCTGCCGTTCTCCTGGTCCTCTTCATGGCCCGCTCCCTGTTGAAAGGTTCAGCAAAGGATGCGAGGTGCCTCGAGCCCTTGGATTTGGGGGATTCCACCTGCTCTGAAGTACGCATAAGGTGGTATTACAACACAGGGAATGAGATCTGTCAAACCTTTCAGTACACTGGATGTGGAGGCAACAACAACAACTTTTACGATGAGAATTCCTGTAAACAATGTTGCGAGTGGGAGTATTCTTGTTCTAAAAGATTTGCACGCCGCTGA

>Lt004

ATGATGATGAGACTGGGCcgCAGCTGCAGACCAGTGGACGTGAAAAGAAAGCATTGGAAACAATCAAACGATTGGACCATGGACATGAAGATCACGTTCAGCGGGTTTGTGCTGGTTGTCTTGGTAACCACTGTCGTTGGCACGTCAGTGCGTCGCGGCGAGGTTGCAGTTAACTTGGTCCATTGTGGGCTGCCTGAGTTTAGGGCGTGTCTCTTGGAGTGTTGGACTTTGAATACAGGCGATTGCTATGCCCGCTGCACTGCAGCGACAGCTGAGTTATTCGGATCACAAACCAATAAATTTGATTGCACAATCTACAAGACTTGCTATTACCGGTGTTACGTGTGCGGAAAACCTGAAGCCCATTGCTGGGAAGGAACCGCAAGTTCAGTGACGGGCAATGTGCACGACCTGGAATCGTGCTGA

>Lt005

ATGGAGGCACTGACTACTCTCAAAGTGTGTGTTCTGGTTGTCTTGACAACCACTGTGGAACTCGCTCCTCCAGCTAACGCGGGTCCAGTGCACGGACTGTGGCGCCGAATCCAGAAACGCCAATGTCCGGTGACCGGGGGCCCCACACCCATGTACCACTGCATGATGGCCTGCATGACCACCAGCACGGAGTACTGGTGCCAACACGAGTACTGCAGCGACTGCGCTGAATGA

>Lt006

ATGGAATTTCGCCGCCTTGTGACGGTTGCGCTGCTTCTGAGTTTGGTCATGAGTATTGATTCAGTGCCTGCCGACGAATTGGAACCTGGAAGAGTTTCCCCGCGCGAGACTGACACATTTCCCTGCTATTCAAACCAATGTGCATGCCTGCCAAAAGGGGGAACGACGACTTCTTACCAGTGCCAGTCGACCACAGCCAGTACAGATAATTGTGTAAATAATGAATGCATAACGGAGGCTAATTGGGGAGGTCGCTAG

>Lt007

ATGTGCGAGCGGTTCATCTATGGTTGGTGTGGAGGCAACCGCAACAATTTCCACAGCTTGCAGGAGTGTCTGGGTCAATGCCTGCCCAAGTGA

>Lt008

ATGGAGAAACTGATAGCCCTGCTTCTTGTTGCTGCTGTACTGATGTCGAACCAGGCCCTGAGTCAATATGGTGAAGTAAAACGCCAAAAGGCGAACACCAACTTTATCCAAAAGAACGTCAGATCCTGGGAGATGGTGGGAGAAACAGCTCGGGAGTTGGTCCACGAATTGTGA

>Lt009

ATGGAGAAACTGATAGCCCTGCTTCTTGTTGCTGCTGTACTGATGTCGAACCAGGCCCTGAGTCAATATGGTGAAGTACAACGCCAAAAGGGGAACACCAACTTTATCCAAAAGAACGTCAGATCCCGGGAGATGGTGGGAGAAACAGCTCGGGAGTTGGTCCACGAATTGTGA

>Lt010

ATGGAGAAACTGACGATACTGATTCTCGTTGCCACCGCACTATTGTCGACCCAGGTCATGGTTGGAGGTGATGGAAAAAAGCCTCTGATGGGGAGGATCAAAGGAAATGCCGCTAAAAGTCTCTCGGCATTCCTGAGAGGGAAAGGCTGCAAAGCACCGAATTCTAGCTGTGAGAGGGATTCCGAGTGCTGTAGCGGCGATTGTCAATGCAGCAATGCTGCTGAGTGCGCTGGCTTTAGTCCAGAGTGTGCGTGA

>Lt011

ATGGAGAAACTGACGATACTGATTCTCGTTGCCACCGCACTATTGTCGACCCAGGTCATGGTTGGAGGTGATGGAAAAAAGCCTCTGATGGGGAGGATCAAAGGAAATGCCGCTAAAAGTCTCTCGGCATTCCTGAGAGGGAAAGGCTGCAAACCACCGAATTCTAGCTGTGAGAGGGATTCCGAGTGCTGTAGCGGCGAATGTAAATGCAGCAATGCTGCTGAGTGCGCTGGCTTTAGTCCTGAGTGTGTGTGA

>Lt012

ATGGAGAAACTTACAATCCTGATTCTTGTTGCCACTGTCCTGTTGGCGATCCAGGTCCTGGTTCAAAGTGACGGAGAAAACCCTGTGAAGGGGAGGGTCAAACACTATGCAGCGAAACGTTTCTCGGCACTCTTCAGAGGGCCACGCGAATGCACAACTAAGCATCGGCGTTGTGAAAAGGATGAGGAATGTTGCCCAAATCTTGAGTGTAAATGCTTAACCAGTCCTGATTGCCAGTCTGGTTATAAATGTAAACCTTGA

>Lt013

ATGGAGAAACTGACAATCCTGCTTCTTGTTGCTGCTTTACTGATGTCGACCCAGGGCCTGATTCAAAGTGGTGGAGAAAACCGCCCAAAGGAGAAGATCAAATTTTTATCAAAGAGAAAATCAGTAGCTGAGAGTTGGTGGGAAGGCGAATGCTTGGGTTGGTCCAATTATTGTACTTCGCACAGCATTTGTTGTTCTGGTGAATGTATTCTTAGTTACTGCGATATTTGGTGA

>Lt014

ATGGAGTCAACCAGCTCAAGTTCCAGCGTAGTCTTCGCAGTGTGGCTGAGGAACGTGTGGGCCGTCGTTGTGGTGGTCTGCGTGTTCTCAATTCTTACTGGGTGTGTGAGGATTCTACCTACAAGTTCTTCGAGGTCATTCTGGTGGATCCATTCCACAAGGCCATCCGACGTGACCCCAAGGCCAACTGGATCTGCAACCCTGTCCACAAGCACCGCGAGATGCGTGGCCTGA

>Lt015

ATGGAAACCGAGGAAAACCTTGGTGAAGTCCGCAAAGTTCCTTGCTGTCTCGGCGTTCGTGACGATTGGTGCTGTGCTGGCCAAATACAAATCTAA

>Lt016

ATGGGAGCAAGGTTTGTTGTAACTGCACTGATTGCCGTGATGGTGCTCTCCCTCATGGTGTCCATGAAGAGCCATAAGCGACGTCTGCCACGCATAGGGAAAAAGAGTTATACTTTTGGTGACTGGAATGCTCGCCAGGGTTTGAGTACTGGTTACAGAGGAACCGGTGGAAACGGGCGGAGTACCGTTTATAAAGGAACCGACGGAAACGGGCGGCAATGA

>Lt017

ATGGGTGCCGTCACTAGAAGTACATGGTGGCGCATTCTGGCAGTGTATGTTCACTCACTCACTCACGCTGGCACCGGGATCGAGCAATTATTGTCCTCACTAGGAGGCTTACTTGTAGGTGGTGAACTGCATATACGTTAG

>Lt018

ATGAGCCGCCTGCCTTGGGCCATGTGTCTGATGCTGCTGATGCTGCTGCTCCTAGGAACTGCCCAGGGCTGCTTCATCAGGAACTGCCCGCGGGGCGGCAAGAGAGCGGTGGACTCCGTGCAACCTACCAGACAGTGCATGTCGTGCGGCCCTGAGGGCGTGGGTCAGTGCGTGGGACCCAGCGTTTGCTGCGGACTGGGCCTTGGATGCCTGATGGGAACGCCGGAAACGGAAGTCTGTCAGAAAGAGAACGAGAGTTCCGTTCCCTGCGCGATCAGTGGTCGCCGCTGCGGCATGGACAACACGGGCAACTGTGTGGCTGATGGCATC

>Lt019

ATGGGGAAGCTGACAATTCTGCTTCTTGTTGCTGCTGCACTGTTGTCGACCCAGGTCATGGTTCAAGGTGGCGGAGATCAACCTGCAGCTCGTAATGCAGTGCCAAGAGACGATAACCCAGATGGAATGAGTGGACAGTTCATGAATGTTCTACGTCGGTCTGGATGTCCGTGGGAACCTTGGTGTGGCTGA

>Lt020

ATGGGCATGCGGATGATGTTCATCATGTTTATGTTGGTTGTCTTGGCAACCACTGTCGTTACCTTCACTTCAGATCGTGCACTTGATGCCATGAATGCTGCAGCCAGCAACAAAGCGTCTCGCCTGATCGCCCTGGCCGTCAGGGGATGCTGTGCCCGTGCTGCCTGTGCCGGGATTCATCAAGAACTTTGTGGAGGAGGACGCTGA

>Lt021

ATGTTGGATCACAACAGGcgCATGGGCATGCGGATGATGTTCACCATGTTTCTGTTGGTTGTCTTGACAACTACTGTGATTTCCTTCAATTCAGATCGTGACTCCAATCACGTGAATCGCAGAGCATCTAAGCGGATGACGCGGGAGATGTGGGATGAATGCTGTGAGGATCCTCCTTGCCGGAACAATCATTTGGAGCATTGTCCCGCACGTTGA

>Lt022

ATGGGCATGCGGATGATGTTCACCATGTTTCTGTTGGTTGTTTTGACAACCACTGTGGTTTCCTTCAATTCAGATCGTGAATCCAATCACGAGAATCGCAGAACATCTAACCAGATTACGCGGGGCGTTTGGGATGAATGCTGTAAAGATCCTCAATGTCGGCAAAATCATATGCAGCATTGTCCCGCACGTTGA

>Lt023

ATGGGGACAATGAAAGCCGCGCTGTTCCTGCTGCTCGTGTTGGCCCTGGGAACTCTTGATGTCAGTGGAGAAGATGGTCAGATGATGCAAGGGAAGAACCCCAGCGATACCTATATTCGGGCTGTAAGACGATCTGCCGGAAAACACAGGTCTAACGTGTGCGCAGGCCTTGAAAGCTGCAGTAATTCTTGCTGCCATGTAGGCAGGAGATGCCAGTGCCTAAAGATACGTTGTGAACAGTAA

>Lt024

ATGGGGACTGTTGCTGGATGGACGTGGTGGCACACTCCGGAGGGAGAGCTCACTCACAATGGCTCCGCAACCAAGCAATTATTGCCGTTAGTAGGGGTATTAGTAGGTGGTGTCCTGTGTACATTGGATCAGAACAGGCGCCACTGA

>Lt025

ATGGGGACTGTCATTGGACGGACGTGGTGGTGCCCTCTGGAGGGAAAGCTCACTCATGCTAGCTCCCCGACTCAGCAATTGTTGTTGTCAGCAGGGGCTTGGAAGGTGGTTTCCTGCATATGTTGGATCAGAACATGCCCTACTGAAAAACACCACTAA

>Lt026

ATGCATGCTGGTTCAATGACTAAGCAGTTATTGTCCTCAGTTTGGGGCTTAGTATGTGCTGTCCTGCAAATATATATCGGGACCACGAAACACCACCACTGTGACTAG

>Lt027

ATGCATCTGTCACTGGCAGGCTCAGCTCTTTTGATGTTGCTTCTGCTGTTTGCCTTGGGCAACTTCGTTGCGGTCCAGTCAGGACAGATTTCAAGAGATGTGGACAATGGACAGCTCACGGATGACCGCCGTAACCTGCAATCGCAGCGGAAGCCAATGACTCTCTCCAGGTCACCTTACAAACGAGCCCTTTGTGGAGAATCTTGCTCATTACATAGTCACTGCATCGGCTTTTGTGAATATTGTTCGGCTAGAACATATACATGCATATGA

>Lt028

ATGCATTTGTCTCTGACAGGCTCAGCTCTTTTGATGTTGCTTCTGCTGTTTGCCTTGGGCAACTTTGTTGGGGCCCAGTCAGGACAGATTTCAAGAGATGTGGACAATGGACAGCTCACGGATGACCACCGTAACCTGCAATCGCAGTGGAATCCAATGACTCTCTTCAGGTCACTTTACAAACGATCCAGTTGTGGAGATCCTTGCGCTCAACATGCAAGTTGCACTGTCACTTGTACATATTGTACGGGTACTCTATTACAATGCATATGA

>Lt029

ATGAAGTTTTTCCTTTCATCTAGGTTGAGGTCCTGTTTGTTCTCCcgAACGGCCTACCAACTCACTACAGTTGAGACTTACTCCAGAGGTAAGTGGATGCATCGTGCTCTGAGGTCAACCGGCAAAAACCCCAAGGTGACCAGAGAATGCTCATCTCCCGATGAATCTTGTACTTACCATTATAACTGCTGCCAGCTCTATTGCAATAAAGAAGAGAACGTGTGCCTTGAAAACTCACCTGAAGTCTGA

>Lt030

ATGCACACACTGGAAATGATGCTGCTCATCCTGCTTCTGATGCCTCTGGCACCTGGTGAGGGAGATGGACGAGCTATGGGAGGAAACAGAAACCCAAGTGAAGCACGCAGTATCCACAAACGCCTTCTGGAAAGACCAGCCAGGCGCCTGGACAGACGCGAATGCACACCCTGTGCCCCTAACCTCTGCTGCGAGCCTGGATCAACTTGTGGAACGTCGTCGACTCACCATGGTTATGGCGAGCCTGCGTGTGTGTATTAA

>Lt031

ATGAAGTTTTCAGTGATGTTCATTCTTTCTCTGGTTTTGACCCTATCCATTGCCGATGGTCTCATTCGCCCCTCGAAAATCGGGGGAAGAACCTTACGGCGGCACAACCCCGATTCAATGGATCTGCAGACGCGTCAGATAAAAACAAAGAATCTGTGCCCTCATTGCCCTGATGGCTGCCACATGGACTTGACCTGCATAGATTAA

>Lt032

ATGAAGCTGTTTGTGGCGATCGTTCTTATTCTGATGCTTCTATCCTTAAGTGCCGGAGCAGAGACGTCTGACAACGGTGTAAGCAGGGGTGGCCATCGACCGCAGTACTGGCCTGTTACTCCGCCGTCAATCGTATGCCTTCGGTCGGGTGAAGATTGCGAGAATAATACCCCATGCTGTCCAGGTCTCTCTTGTCGTGTGTCGGCCGATCTTGCTACACTGAAATTATCACTTGCCTGCGACTAA

>Lt033

ATGAAGCTGTCAGTGATGTTCATTGTCTTTCTGATGCTGACCATGCCCATGACCTGTGCTGGCATTAGTCGCAGCGCTACCAACGGGGGAGAGGCCGATGTGCGAGCACATGACAAGGCAGCTAACCTAATGGCGCTCCTACAGGAAAGAATGTGCCCTCCCTTGTGCAAACCCAGCTGCACAAATTGCGGCTGA

>Lt034

ATGAAGCTGTCGGTGACGATCATTCTTGTTCTGATGCTGACCACGTCCTTGACCTGTGGATTTAACCACTTCTCTAACAATGGGAAGAGGGCCTATGGACGACATGACCCCAATGCGGCGGATCGATTGGTTCGTGAGAAACAAGCAAGCCGCGCGTGCTATCCGCCCTGCTTTGGTTCATCAGTATGCTATGGTGGAAGATGTTTCTTTATAGGTTTCCGGTAG

>Lt035

ATGAAACTGACGTGCATGATGATCGTTGCTGTGCTGTTCTTGACTGCCTGGACATTTGTCACGGCTGATGACCCCAGAGATGGACTGGAGGATAGAGGAGGATGGGGGCAGGCAGGAGGATGGGGGAAGCTTTTCTCGAAGGCACGTAACGAAATGAAGAACCCCAAAGCCTCTAAACTGGACAATACGGAAAGGCGCATCTAA

>Lt036

ATGAAACTGACGTGCATGATGATCGTTGCTGTGCTGTTCTTGACTGCCTGGACATTTGTCACGGCTGATGACCCCAGAGATGGACTGGAGAATAGAGGATTAACGGGGGAGGCAGGAATGTTGGAGGGATTGTCCTCGAAGGCACGTGACGAAATGAAGAACTCCGAAGCCTCTAAATTGGACAATACGGAAAGGCGCATCTAA

>Lt037

ATGAAACTGACGTGCGTGTTGATCATCGCCGTGCTGTTCCTGATGGACAATCAGCTCATTACAGCTGATTACTCCAGAGATGAGCAGGTATACCGTGCAGTGAGGTTGAGAGACGCAATGCAGAAATCTAAAGGTTCTGGGTCGTGCGCGTACATAAGTGAACCTTGTGATATTCTCCCTTGCTGCCCTGGTCTGAAGTGCAATGAGGACTTTGTACCCATATGTCTCTGA

>Lt038

ATGAAACTGACGTGCGTGCCAATTGTCGCCATGCTGTTCCTGATGGCCTGTCAACTCATTACAGCTGATTACTCTAGAGAGAAGCACGGGTATTCTGCTGAGAAGTCGAGTGACAAGATTCAGGATTCCTTTTACTCAAAGTTGACCAAGAGATGTACGGATGAGGGTGGTGATTGTGATCCTGGAAATCATAACTGCTGCAGGGGATCGTGTCTTGTTTTACAACACAAAGCCATCTGTGGTATAGTTTATACTATGGTCTCGCGGTAA

>Lt039

ATGAAACTGACGTGCGTGCCGATTGTCGCCATGCTGTTCCTGATGGCCTGTCAACTCATTACAGCTGATTACTCTAGAGAGAAGCACGGGTATTCTGCTGAGAAGTCGAGTGACAAGATTCAGGATTCCTTTTACTCAAAGTTGACCAAGAGATGTACGGATGAGGGTGGTGATTGTGATCCTGGAAATCATAACTGCTGCAGGGGATCGTGTCTTGTTTTACAACACAAAGCCGTCTGTGGTATACTTTATACTATGGTCTCGCGGTAA

>Lt040

ATGAAACTGACGAGTGTGGTGATCGTCGCTGTGTTGTTCCTGGCGGCCTGTCAACTCACTACATCTGATGGCTCCAGAGGTACGTGGAAGGATCGTGCTGTGAGGTCGATCACCAAAGTCTCCATGTTGCGATGGCCCTGCAAGGTTGCCGGTAGTCCTTGTGGTCTTGTTAGTGAATGCTGCGGAACTTGCAATGTGTTACGCAATAGATGTGTGTGA

>Lt041

ATGAAACGTACGTGCGCTTTGATCGTCGTCCTGCTGTTCCTGACGGCCTATCAACTCACTACAACTGATGACTCCAGAGGCAGGAAGGGGTATCGTGCTGAGAGGGCGAGAACTAGGATAACGAATTCCAAGCTCCTCAAGTTGACCAAGAGGTGTGTTGAACCCGGCTCCCCTTGTTCGAAGTATGACAATGAATGCTGCGATGCATGTATGTTAAGTCATCCGAACCCTCCGGTTTGCATAGAGTGA

>Lt042

ATGAAGGTTGTGGCTGTGTTTCTGGTTGTCGCCCTTGCTGTGGCTTATGGCCAGTTCTTCTGCCCGGACAGCGAAAACGATCCCCTGAACTGCCTTGAGACCATGGCGTCCGCCGCCACCTGCATGAAGTCCAAGTCGGATGGCACCTACTCCTACGCGTGCGGCTACTGCGGCAAGAAGAAGGAGACTTGTTTCGGCGACAAAGTGCCCGTGACGGACTACAACTGCCAGAGGCACAAGATTGTCAACCACTGCGGAGGCCCTGTTGTCTGA

>Lt043

ATGAAGGTTGTGCTTGTTCTACTGGCTGTACTGGTGGCGGCCTCCGCCGCGCCTCAGAAACGGTTTTTCCTTCATGACATCCAGAACTGGATCCATCAACTGCAGAACGCTATTAACAAAGCCAAGGACAAGTTTAACGAACTCACTTCTGGCCTAGGGGTCCACTTCGACAGGATTGTCGACCTGCTGGTCGACCAGATTGATTCGACCATGACGGAGGCCGCCTGCATAAAGCTGTGCGAGGGCAGCGCCAGCAAAATTCTTGGACAGGCTTCCTCCATGGCCGGCACGGTCTGCGCTCCAGTCTGCACAGCTGCCCTGGCGAAACTGGAGGAGGCCGCTGGTTAA

>Lt044

ATGCTGTGTCTTCCGGTCTTCATCATCCTTCTTCTGTTGGCTTCACCTGCAGCTCCAAAGTCTTTGGAAACGAGAATCCAGAACGATTTGATTCGCGCAGGCCTTACAGATGCCGATCTGAAAACCGAAAAAGGCTTCCTTAGCGGCCTACTCAACGTGGCCGGCAGTGTGTGCTGCAAGGTTGATACCAGTTGCTGTTCTAACCAATAA

>Lt045

ATGTTGTTTATTACGAGGTGGTGCTGCACTTCATGCAAAATGCCGCCATGCACATGCTGCTATTAG

>Lt046

ATGTTGAAAATGGGAGTGCTGCTGTTCACCTTTCTGGTCTTGTTTCCCCTGACAACGCTCGAACTGGATACAGATCGACCTGTAGAACGACATGCGGCAATCAAACAGGACCTCAAACCACAGGAAAGGAGGGGAATAAGATTGCACGCTCCAAGGGACGAATGCTGTGAACCGCAATGGTGCGACGGAGCTTGTGACTGCTGCTCGTAG

>Lt047

ATGTTAAAAATGGGAGTGCTGCTATTCACCTTTCTGGTCTTGTTGCCCCTGGCAATGTTCCAACTGGATGCAGATCAGCCTGTAGAACGATATGCGGAGAACAAACAGGACCTCAACCGAGATGAAAGGATGAAAATCATGTTGTCTGCTCTGAGGCAGCGGCAATGCTGTGATTGGGAATGGTGCGACGAACTTTGTTCTTGCTGCTGGTAG

>Lt048

ATGTTGAAAATGGGAGTAGTACTATTCACCTTTCTGGTCCTGTTTCCCCTGGCAACGCTCCAGCTGGATGCAGATCAACCTGTAGAACGATATGCGGAGAACAAACAGGACCTCAACCCAAATGAAAGGATGAAAATGATAATGTCTGCTTTGGGGCAGAGGCGATGCTGTATTTCGCCAGCGTGTCACGAGGAGTGTTATTGCTGCCAGTAG

>Lt049

ATGATGACCGTGGACACCAAGGGAAAAGGTCTcgTCAAGTCAGCTTTACACACCAGCATCACTGTGCGATACAGTGCGGATCACCCGAACCTGATCAACATGTTGCGACTGATAATCGCCGCCGTCCTCGCCTCCGCCTGCCTCGCTTACCCTGCAAAAAGAGATGGTGCCCCAGCAGATTCGGCCAACCTCCAGAGCTTTGGTCCGGGAATGATGCAGGCTATGCCCAACATGCAGGGAATGCAGCAGCCAATGCAGGCAATGCCCGCTGGTCAATTCATGCCTTTCAACCCTAACCTTGGCATAGGATACAAGAGGGCTGTCTATGAGGACCGAGAGGAGAGGAGACCGCACTCTAAGTTCAACGACAACAAATCTCCCTTTGAACAAGAGGGTGGCCTAGGAAACTTCATGAACTTCATGAAGGAAAACGGCAACCTTCCATTCGCAAACATGGACGGCGCCGCTGCTGACCTTGGCAAATTCGAGCCCAGCGCCGAAAAAGGGCAGAAGGAGGGCCAGTTCCGCTTCTTTGACAAGCAACAATAA

>Lt050

ATGCTTTCCACCTGCGTGTCCGCTTACACCCTTTCCACATCATCCGCATCAACAAGATGTTGTCCTGTGCTGGGGCTGACAGGCTCCAGACTGGTATGCGTGGTGCCTTTGGAAAGCCACAGGGCACTGTGGCCCGGGTGCACATTGGGCAGCCCATCATGTCTGTGCGTGCCCGTGAGCAGCATGAGGCTTCCATCATTGAAGCTCTTCGTCGTGCCAAGTTCAAGTACCCAGGACGCCAGAAGATCGTGGTGTCCAAGAAGTGGGGATTCACCAAGTGGCCACGTGAGGACTACGAGCGCATGCGTGCTGAGGGCTACCTGA

>Lt051

ATGATGTGTCGTTTGACGTCACTCTGCTGTTTGCTGGTCATCGTTCTCCTGAACTCGGCTGTAGGTGGGGGTATTCCCTGTACCGGGTCGGGAGGATTCTGCTCTTCGTATATGTGGTGCTGCAATTCGTTTGATGTCTGTTGTGAAGAATTCCCCGGACCGGCGAGGTGCATGAGCGAAAGTGCGTGTTCATCTATAGAACGCTGGGCACAATACACCCATTTCTTCAGACGTTGA

>Lt052

ATGATGTTTCGTTTGACGTCAGTCAGCTGTTTCCTGCTATTCATCGTTTTTCTGAATTTGGTTGTGCTTACCAATGCCTGCATCCCTGAAGGAACGTTCTGCCAATTTAATGCTGATTGCTGTCTATCACAATGCTGTTGGGGGAGTTGCGGAAATCCGTGCCGCTTTCCTGGAAAGAGGGAGAAACTCCAAGAATTCTTTCGACAACGTTGA

>Lt053

ATGAAAAACCTCTTGCATCTGGACACTACTGTTTCGACAAAGTCGGTGAGAATAAAACTCCCAATAGTGAAAGCCCCcgCGCCTCTGTTCCATCAAACTTTGCACAGCTTCACATCACGTTGTGGACGTCAGAAGTGGGTGGGCACGCCAACTGTCAAGGGTCTTGCACCATTCAAAATTAAAAAACACAAAACTCTTGGAAGCAGACAGACAGTTCTGGTGGTCATGATGAAACTTTTGATGACATTCGTTCTTCTCCTGATGATTCTGCCCCTCTGCCAACCCTCAGGGCGCAGACAACTGCCAGCCATGCCATTTCCAGATAGCTCAATTACACCGAAGCCTACTGTGCCGCCGGACTGTGCTTTCAAATCGTGCCAGACAAAAGCTGAATGCTGCAACGGATATTACTTTTGCGGTGCCGGATTTTGTATTCCCAATATGAATAAAATATTCGATTATGGCAAATAG

>Lt054

ATGATGTTGGAACTGAGAGTTGTGCTGTACATCCTCCTGGTTCTTTTGCCCCCGACAACCCTTCAAGAGAAAGAAGAAGATGACACCTCTGATGCACTGGTCAACCAGTTGGACGAGAAGATTGCAGACGGCACTATCTTGGTGCGCGACCTGAGCAGAGATGCAGATGACGTCCCTTGTAGCACCAACGGATGCAATGGAAATCACAACTGCTCCACCAATTGCAATTGTACCTTCAACATGGCTTCGCGTTGCCTTAATTGCGTATGCTAA

>Lt055

ATGATGCTGTTTATGTTCGCTGCCATCATCTTCACCATGGCCACTGCAACAGTCAGTGCGACCCAGTGTAATAGTGTTCAGACACTGTGCTCATGGGAAGGAATGGGTGAACTCTGCAACTGCTCTGCAACTGGCTCCTGCCCCCAGAATGATGAACACAAAATTGTAGTTGGCACCCAGGACATTTACGTGTGCCAGCAGATCTCTGACTTCGAAGTTTGTACGGGCTCGGTTGCGATCGACTCTTCCCTGACCGAGTTGAACTGCAGATGTTCAAGTAATGCATACAAAATAGAAGGCTCGCAGGTTGTATGTGACTAG

>Lt056

ATGATGCTGTCTTCATACGTACCCGTCTACATCGCCGCCCTAACGACAATGATGGCGATCAGTGGTGAAGCATCCTCCTCCATCTCTTTTTGGAATAACAATGCCACGTGTATAGAAGACGAGACTTGTGGATACAAATACTCCTCGTTTGGCCTTGGCGTTCAGTATTGTATGTGTCCTGGAAGCATACTCTGTGACATTGACGGCCCTGCTACAGCTGTATTGCTTGGAGAAAATAAAACGTATTACACCTGTGAAGCGCCCACAGACTATCAGGAGTGTGGTGCGAACGAGTATCCGACTGAAATAATAATAATGGAGAACCGGAATCTAAATAGAACCACCAAGTTTGTTTGCAGATGTCCAAATGAACATCATCGATTCAATCAGACTCTCCGTCGTCTTGAATGTTACTAA

>Lt057

ATGATGCAGAGAGGGGCCGTGCTGCTCGGGGTGGTCGCCTTCCTTGCCCTGTGGCCCCAGGCCGCTGCCAAGGTGTACAATTTGCACGAAACTGAAGTGTGGGCCGTGGTCGCCTATTCAAAGAGAGTGATGCACGCCTGTGCCATAGCCAATAGCCACATGGACGACCCATGGCTTGTGGTGGATGTTAAGGACTTTGAAGAAAGGAGTCTATTCCATTCAATGTACAAGGCAATGGTATCCTGCCTGGAAGATTTTTTCCAGCAAAGACCATGA

>Lt058

ATGATGTCAAAACTGGGAGCCTTGCTGACCATCTGTCTGCTTCTGTTTCCACTTACTGCTGTTCCGCTGGATGGAGATCAACCTCTAGACCGACACGCGGAGCGTATGCATGATGGCATTTCACCTAAACGCCATCCCTGGTTTGATCCCGTCAAACGGTGTTGCAAGGTGCAATGCGAGTCTTGCACCCCTTGTTGCTAA

>Lt059

ATGATGACTGTCGCGTCGCCTTGCCACATCTTGCCTGGGGGTAATGCGCTGAAATTTGGTTTCGCACCAATCATTTCACTATAG

>Lt060

ATGATGACTGTCGCGTCGCCTTGCCACATCTTGCCTGGGGGTAATGCGCTGAAATTTGGTTTCACATCAATCATTTCACTATAG

>Lt061

ATGATGACGAGAAGAAAAGGAAGAcgGCTCGGGGTGGTCGCCTTCCTTGGCCTGTTGCCCCAGGCCGCTGCCAAGGTGTACAATTTGCACGAAACTGAAGTGTGGGCCGTGGTCGCCTATTCAAAGAGACTGATGAACGCCTGTGCCATAGCCAATAGCCACATGGACAACCCATGGTCTGTGGTGGATGTTAAGGACTTTGAAGAAAGGAGTCTATACCATTCATTGTACAAAGCAATGGTATCCTGCCTGGAAGATTTTTTCCAGCAAAGACCATGA

>Lt062

ATGTCTATAGCGTGCAGTCTGTTTTGTGTATCTCTATGGATCCTTTACAGAGAGTCTGTTTTGTGTATCTCTACAGcgAGAGAGAGAGAGAGAGAGAGAGACCTTAGGAGTTGTGTATTCTCCAGCCATCTGGCGGCCCCATCCACGTCGGTGCTGTTTCGTCTGTCACCCAGTCCACATCATCGCTCTACCATCTGTCAGCCTGTTTGCATCAGCACTGTGATCTCAGACTGTCCAGTTCCCCCCAGAAATGTTTCACAAACTGACGCTGCCACAAGTAATGTTGAACTGAGTTGGTGTCGGATTCACAAAATGTACACAGGTTGTAAAATGGAGGGACGTCGTTTTGCTGCTGTTCTGATCCTGGCCATCTGTATGCTTGCACCTAGGGCTAGTGCTTCGAGACCTCATGTTCTACGGAGAGTATGCAAACAACCAGCGGAAACGGGGCGGTGCTGTCGGGTTATCCAGAGATATTACTTCGATATGGATTCTTACGACTGTAAAAAGTTCAATTACAAAGGATGTGGTGGCAACGGCAACAATTTTCGCACTTACAATGAGTGTTACGTCACATGTGGGCGGAAATATGTGAACTGGTGGTACTGGCGACATCCGTAG

>Lt063

ATGACGACTGATGATAGGATGTCTGCCGCTACACACAGGCcgTTGCAATCCCTTGTTCGTTGTTGCTTACCAGGAGGCGGGATGCCGTCGGTGAGGTGGCTGTTGGCGGTGACCACGGTGGCCGTTGTGGTGATCGTGATGGTATTGAGTGGCCCTTACTCCGGTTTCCGGACCCCTAACCAGGCCACGGTTCAGATGACCAGGAATGCGGGCCATCACGAAGGGAAGGCAACCAAACTGCAGACCGGTGGAACATCTTCAGGCATTGTTGACAGGTGCCGCCTGCCCCCAGAGACTGGCATGTGCAGAGCGTACATACCCATGTATTTCTACAACGCCACGCTGGGTCGGTGCCAGGGGTTCATCTACGGCGGCTGCAACGGCAACGACAACAAGTTCAACACGGAGGAGAACTGCATGAAGGCGTGCCATCATTGA

>Lt064

ATGAATAACTTTAATATGTCAGTACTGTCTTATATGGTTCATATGTTGCTGAAATTGCCATGGTTATTTCGCAGTCACAGGTTGCAAAAATTTTGTGAAGATCACAACTTTGTGTACCACTAG

>Lt065

ATGAACAACACTGGCCGACTCCTGCTTCTGTGCCTCGCTCTGGGCCTGGTGTTCGGGTCTCTGGGAAAATTCGTTACTGATAACGGAGACGCTGACAGAGACGCAGGTCCTGTCAAGAGGGCAGCGTTCATAGCTGACAGGATGACCGAGGTGGATTGCGGCGGTGTATCATGTGAATTCCACTGCTGTGAAACAATCAACGGGGTACAGAGATGCCGAGAAATCAACTGTAACTGA

>Lt066

ATGCCCTGCTGTCATGGTGACCTCAGTTTCAATTGCCTGTCCATGTCTATGGCTTTGGTTTGGTTCTGTTGGTATCACCAGTACAGGCAAATTCATGGGGACTGTCGTTCGGATGGAGGTGCGCCGCATTTGGGGGAGGGAGAGCCCAGGCATGCTGGTTCCATGACTAAGCAATTATTGTCATCTATAGGGGGCTTAGCAGGTGGTGTCCTATGCATATTGGATCAGACAAGGCGCCATTGA

>Lt067

ATGCCTAAACTGGGAGTCTCGCTGTTCATTTTTCTGGTTCTGTTTCCCCTGGCAACTCTTCAGCTGGATGGAGATCAATCCGCTGGTCGTCATGCACAGGAAAGGGGCGAGGACCTGTTTAAAATGTATCAATACTTGAGGCGCGCTCTGGAAAGGAGGCGTACTGGTGAGGACTTTCTGGAGGAATGCATGGGTGGCTGCGCCTTTGACTTCTGTTGCAAAAGGTCTTTGAGAGACACTACATCGGACTGA

>Lt068

ATGTTTAAAAAGGCAGGTGCAGATAAGACCCTCACCACCACGTCAGCAGAGAGAGAGAGAGAGAGATACCTTAGGAGTTGTGTATTCTCCAGCCATCTGGCGGCCCCATCCACGTCGGTGCTGTTTCGTCTGTCACCCAGTCCACATCATCGCTCTACCATCTGTCAGCCTGTTTGCATCAGCACTGTGATCTCAGACTGCCCAGGTTGTAAAATGGAGGGACGTCGTTTTGCTGCTGCTCTGATCCTGGCCATCTGTATGCTTGCACCTGGGGCTAGTGCTTCGAGACGTGACGTTCTACTGAGTGCATGCAAGCTACCAGCGAAAACGGGGCCGTGCTATTCGTTTGTGCGGAAATATTACTTCGATTGGGATTCTGTCAACTGTAAAACGTTCATTTACGGAGGATGTGATGGCAACAGCAACAATTTTTGCACTTACAATGAGTGTTACAGCAGATGTGGGCCGGTAAATCGGCGCTGA

>Lt069

ATGGAGGGACGTCGTCTTGCTGCTGTTCTGATCCTGGCCATCTGTATGCTTTCACCTGGGGCTAGTGCTTCCAGACGTCATGTTGTGAGTTTATGCATGCTACCGGCGGATACGGGACCGTGCTTTGCGTCTATGCCGAGATATTACTTCGATATGGATTCTTACGACTGTACAACATTCGTCTACGGAGGATGTGATGGCAACGGAAACAATTTTCGCACTTACGTTGAGTGTTACGGCACATGTGGGCTGGAATAA

>Lt070

ATGCAATCCTTCACCTGCATGTGTCTGCTGGTTCCACTCCTCCTCTTTTTCCATCTGACTCAAGTATCGGACACGGCAGACCATGGAGGGTCCGCCACGGACGTCCGCTCGGCTGACCGCACCCTGAAACGATTTCCCCATGACTTCCGCCGGGCCCCGAAACGAAGATCCGACGGTCACTACGATAGGAACCGGAAAACCTCTGTGCAGTTAGACACGGAATTATTGGTTGAGGGAGTTCGCGAGGGTCAAGAGGAGAGAGCTGAAGCTTCATATGAAAAGCTGCTAGAGATAGGTCGTTGA

>Lt071

ATGAGAGCTTCCACCTGGTTGTCGGGTAGAATGGTCATCACCGTCTTGCCGTCCTTACGGGTCAGCGTGGCCATCTCCACTTTGTCTGGCGTCAGCTTGGTCAGGTCCAGGCTTTTGCTCAGCACCTTGACAGCGAGGGCCAGGGCCTCATCCAGGCTGGTCTCCCCCTCCTTGTACTCCTGCTTCAGCAGGGACACTGCGTTAGCGCTGTTGTTGCCGATGCATGTCGCCTTCCAGCCGCCATAG

>Lt072

ATGCGCTGTCTCCCAGTCTTCATCATTCTTCTGCTGCTGATTCCATCTGCACCCAGCGTTGATGCCCAACCGATGACCAAAGATGATGTGCCCCTGGCATCTCTACATGATAATGCAAAGCGAGCCCTACAAATGTTTTGGAACAAACGCGATTGCTGCCCAGCAAAACTTTTATGCTGTAATCCATGA

>Lt073

ATGCGCAAACACACACAGACAAAAAAAAAAAAAAAAACAACACTGGGAATGGTACTACTGCTCCTTCTGGTTCTCCTGCCCTTGGGAAACTCAGATGGTGACGGAGATCGGCAAGCGATGGACAGAGACCATACTGCCAGTAAGGAACGCGGTGCCCCCATACTCCGTCTGAGAAGACACATGGACCATGGCCGATCTATCAACAAACGTTGCTCCACTAAGATGTGCGGTGACGACTGCTGCCCTAGCACTTCCTGTGAGTGCGATGTTGTTGGTTCACAGGGCAATGAAATGGGCTGCAGCTGCCCCGTCCTACTCCTCAGAAAGTGA

>Lt074

ATGCGCCTTTCCGTATTGTCCAGTTTAGAGGCTTTGGGGTTCTTCATTTCGTTACGTGCCTTCGAGAAAAGCTTCCCCCATCCTCCTGCCTGCCCCCATCCTCCTCTATCCTCCAGTCCATCTCTGGGGTCATCAGCCGTGACAAATGTCCAGGCAGTCAAGAACAGCACAGCAACGATCATCATGCACGTCAGTTTCATCTTGGTGATGTTTTAG

>Lt075

ATGCGGAATTTCAGAGGTTTTAGGTCGTGCAGTGAACAAGGTGAAGGTTGTGCTACTCGCCCTTGCTGCCCTGGTCTGAACTGCGTTGGCGGCATTGCTGGAGGCTTATGCCAGGACTAG

>Lt076

ATGACGTCTGTTCAGTCTGTGACCTGCTGCTGCTGCCTGCTGTGGCTGATGCTCTCTCTCACTCTCGTCACTCCTGGATCCTCTGGACCTGCACAGCGGCCTGGGCATCGCGCTGCCAGAGTTCCTGCAGAGCCGATATTGGAACAGCTATGCCCTGAAATGTGCAACAGGGGAGAACTGGAGTTCTTTTGCACCTGCGGATCAAGACAGTTCGTGGTTACTCTACCGGTCATAGAGCGAAAAAGATCGATGGCGGTGTGA

>Lt077

ATGAGGACCAGCGGCCGACTCCTGTTTCTGTTTCTCGCTGTGGGACTGCTGTTGGAGTCTCAGGCACACCCCATTGCTGATACTGGAGATGCTACCAGAAACGTGGGTTCTGACGGAACATCAGTGGAGTTGTCAGAAATGCTTGAAAAAGGGAGAGATTCGTCAGCTGAGGAAGGACTGAAAAAAGCCAGTGATCATGGTCATGATGATGACCACGTTGACCCTGAACCTGACCATGACATACCTTTTCCATAG

>Lt078

ATGTCTGGATTGGGAATCATGCTGCTTGCTCTTCTACTTCTCGTGTCCCTGGAAACCAGCCTGCAGGGCAGAGGAGGAGAGGGACAGGCGGTGCTTCCTGACAAGAACCAACAAGGAAGCAGAAAAATACTCCTGAGACGTGCTCTCCAAAAATTACGCAAACCTCCACGAGGGACCAAGAAGTCTGATTCTCTTGATATCCTGGTAACTGCATTTGTCGCTTAA

>Lt079

ATGTCAGGATTGGGGATCATGGTACTAACCCTTCTACTTCTGGTGTTCATGGCAACCAGTCATCAGCACAGAGGAGGGAAACAGCTGATGCTTCGCAACAATCTCCAAAAGAGAACCTGCAGTTCTCCTTCAAATTGCCCCACGGGTCAAGAATGCTGCCCCGACAAAGTGGACGAACCCGAGGGGTTCTGTGCTGACGAGTGCATTATCACCTAA

>Lt080

ATGTCAGGATCTGGAGTGGCACTGATCACCTTCCTGCTTCTGTTGTCCCTGATGACCAATCTGCAGGGTGGAGGAGAGGGACAGAGGATGCATCAAGACAAGCACCGACAGACAGCGAGGAAACTGTTTACCTTGGGTCGTAAAGTGAAAAGAAACGACCCTTGCGAACTCGATTCTCCCTATGACGACTGTACTGGCACGCAGATATGCTGCACTCAACCAGGGTCGATGAGCGGGGAGTGTAAAGAGGCCGACGAATGCTAA

>Lt081

ATGACAAGTAAAAAAAAAAAAAAAAAAAAGAAcgTGTGTGTGTGTGTGTGTGTGAAAGTGTACACGTGGCAGCGGCCCCACAAAGCGACGCTACGCAGTCCTAACCCTTTTCTTTTTTTATGCATTTTCAGTTTTGACTTCTGCTCTCTCTGTTTGCCTGACAGCTGCTCCGAACTTCACAGCCATAGTCTCAGCGTGGACCTGATACAGAATATCGCAACTGCCAAAACTCTTGTTGCACCGTACCTTGACGTGACGTCATCATTTCAGCACTCTCTGCTTAGCTCCCTTGGCAACATGTCACACCTCTTCCTGGGTCTTCTGGTGATTTCTGTCATCATTCTCCACACTGACTCATCCCAGGATACTGACGGTAGCGTTGACGTGGGGTCCAGACCTTTGTCACGTGCTGTCACGGGTGATGGGTTTCAGCGTAAACTACGGGTCCGCATCTTCCGAAGAGCGGCCTTAGATGACTTCACGGGAACAGAGCAGGAAGAGGAGGAAGACGAGGAAGATGAGTGGTTTGAACGTCGACAAGCTGTGCAGAAAAGGGGGATTCCAAGAATGGAAAAATGGAACATCAACAAGAAATATTGA

>Lt082

ATGTCACACCTCTTCCTGGGTCTTCTGGTGATTTCTGTCATCATTCTCCACACTGACTCATCCCAGGGTATTGACAGTAGCACTGACGTGGGGTCCAGACCTTTGTCACGTGCTGTCACGGGTTTTGGTTATCAGGGCAAACTACGGATCCGCGCCTTCCGAAGACATGTTTTGGAAGACAAAACGGCTGCAGAGCAGGAACAGGAGGAATGGGAAGAAGAATTTTACTGGTTGTTATTTCCATAA

>Lt083

ATGTCTAAACTGGGAGTCGTGTTGTTCATCTTTCTGGTTCTGTTTCCCATGGCAACACTTCAGCTGGATGGAGATCAACCCGCTGATCGTCGTGCAGATGAAAAGGACCTAACACAACAGTATCTAAACTTGAGGCGTGTTCTGCAAAGGGGTTTGGTATGCGCTCATGCAAGTCCATACCACAATGCCGTCTGGTCGTGA

>Lt084

ATGTCTAAACTGGGAGTCGTGTTGTTCATCTTTCTGGTTCTGTTTCCCATGGCAACACTTCAGCTGGATGGAGATCAACCCGCTGATCGTCGTGCAGACGAAAAGGACCAGGACCTAACACAACAGTATCTAAACTTGAGGCGTGTTCTGCAAAGGGGTTTGGTATGCGCTCATGTACGTCCATACCACAATTCCATGTGGTCTTGA

>Lt085

ATGTCTAAACTGGGAGTCGTGTTGTTCATCTTTCTGGTTCTGTTTCCCATGGCAACACTTCAGCTGGATGGAGATCAACCCGCTGATCGTCGTGCAGACGAAAAGGACCAGGACCTAACACAACAGTATCTAAACTTGAGGCGTGTTCTGCAAAGGGGTTTGGTATGCACTCATGTACGTCCATACCACAATGCCGTCTGGTCGTGA

>Lt086

ATGTCAAAACTGGGAGTGGTGCTGCTCATCTTTCTGGTTCTTTTGCCTCTGACATCTCCTCATCAGAATGGGAATGGATTTGCTGGTAACCAGGCGAGGCAAATGGGCGTGCAACGAAGAAAGATTGGGTTGGCCAATGCTCTGAGACGTTCAAGCTGTGGATACCTGGGGCAGCAGTGCTGTATCGTTCCCAAACGTGCGTATTGCCATGGGGACCTTGAATGCAACCCCGTAGCAATGTGCGTTGCGTAA

>Lt087

ATGTCCAAATCAGGAATGGTGCTGTTCGTCCTTCTGCTGTTGTTGCCCCTGGCATTCCCTAAACTGGTTCCGGCTGGACGTTCAGTGGCACGTCGCTACGGAAACCTTGGTGCAAAGCGCGAGGTTCCACTTACTTGTCACCCCCCGTCAACCCCAAATCTGAATGGCCCGTGGCAAGATCAAAAGTGTTGCCTTACCAAGCAGTGCAGTCCAACAAACTGCTGTACCAGCACGTCCTGTGTTTGCGATATGTCAATATGCGACTGCTCTGGCATGTAG

>Lt088

ATGTCTCGCCTCTTCCTGGTTCTTTTAGTGATTTCCGTCATCACAGTGTGGACTAGTGCTTTCCAGGATGGTGATGGTGGCATTGTCAAGAGGTCCGGACAGTTGTCACCTGCTATCGAGAACACCGCCTACACTGGTTCCTCCTGGAAACATCAGGACACCACCAAGTTTCGCAGATTTCGACGTGGAATGGGTGACGATACCATAATAGAAGAGGTATTAAACGAAGAGCCTCGGCGGACTGACTTCGTAGAAAACAAAAATAAAAAAGAATTTGACAAACAAGTGAGAGAGAATTTACGACATCTGTAA

>Lt089

ATGTCACGCCTGATCCTGGTTCTTCTGACGATTTCCGTCCTCACTCTCCACACTGACTCCACCCAGGGCCATGATGGTGGTACGGACAAGAGCTCCAGACCTGTGGCACGTGCTGCCGGAGACCACGCCTCCCCTGCACTCTTCCGCAAATTCCGGGCCCGCGCCAACGTCCGCACCCGGAAAATTGGACGTGCTGTTGAAGACTATCCCGGCGGTGAGGAGGAAGAGAATGAGGAGTGGGGCGATGATAGAAAGGCTCTGTGGGCCCAAAAGATTCACAATACGTTTTTCAACCCCTACCGCCTTCATTGGCCGACCTTTTGA

>Lt090

ATGAGTTCAATTTCCAAGACACTTAGTCTAATTTGTTATGTAGTTCTGAAAATAAAAAAAAGGAAAATATACTGCATAAGACAGCGTGTATTTTTACGCCCATTTCAATCCACACGAGCACGTGCAAAAGAAACGGTCTATTTTTCACATCTCATTTCCGGAAACAGTTTGCGGGTGTCGTTGCCGAAATCAGTCATACTTTAG

>Lt091

ATGTCAACACTGGGAATGGTGCTGTTGCTCCTTCTGGTTCTCCTGCCCCTGGGAAACTCAGATGGGGACGGGGATCGGCAAGCGATGGACAGAGACCATACTGCCAGTAAGGAACGCGGTGCCCCCATACTCCGTCTGAGAAGACACATGGACCATGGCCGATCTATCAACAAACGTTGCTCCACTAAGGTGTGCGGTGAAGACTGCTGCTCTAGCAGTTCCTGTGAGTGCGAAACTGTTGGTTCACAGGACAATGAAGTGGGCTGCAGCTGCCCCGTCTTCGGAAAGTGA

>Lt092

ATGTCAACACTGGGAATGGTGCTGTTGCTCCTTCTGGTTCTCCTGCCCTTGGGAAACTCAGATGGTGACGGAGATCGGCAAGCGATGGACAGAGACCGTACTGCCAGTAAGGAACGCAGTGCCCCCAGACTCCGTCTGAGAAGACACATGGACCATGGCCGATCTATCAACAAACGTTGCTCCACTAAGATGTGTGGTGAAGACTGCTGCCCTAGCACTTCCTGTGAGTGCGAAACTGTTGGTTCACAGAGCAATGAAGTGGGCTGCAGCTGCCCCGTCTGA

>Lt093

ATGTCGACCCAGGGCCTGATTCAAGAAAAACGCCAAAAGGCGAAGATCACCATTTTTTCAAAAAGGAAGTCAAATGCTGAGAGGTGGTGGGAGGGCGATTGCACTGATTGGTTAGGGTCGTGTTCATCGCCCTCGGAGTGTTGTTATGACAATTGTGAAACGTACTGCACGTTGTGGAAATGA

>Lt094

ATGACCATTTCTCTATTGTTTAAATACATCATTTTAACAGATGGTCTCAAACATGAAAGATTGAACATACATATATTCTATCCCTTTACTGCCGCCCTCAATTCAATAAAACCGGATAAAATGAAAAACATTGGGAAAAAAAAAAAAAAACACAGCAGACTTGCGGTTATTTCATGCAGACATTCTGAGACCATGTTTCTGCTTAACGTTGCTGACTCCCTAACAATTCACACTGACTTTATTGAAATATGGGGAGTTTGTAGTATACTCCCTGTTTGGCTAGTACACTTGCCATGTCAGACCATAGCATTCAACGGCAGTTAG

>Lt095

ATGACTCTCGCATCTTCACGAGATTTTGCAAAGAAAACCCCGAAAAGACTTGCAAAACTACGTGACTGCTGCCCCAGAAGTATCTTATGCTGTGAGTTTTCATAA

>Lt096

ATGACTCTCTTCAGGTCACTAAATAAACGATCCAGTTGTGGAGAACCTTGCATACAAGATGAGCATTGCAGTGGCACTTGTAATATTTGTACGGGTAATCTATCAAAATGCATGTGA

>Lt097

ATGACTCTGACGAAATCAGCTGTTCTGATTCTGGTGCTACTGTTGGCCTTTGACAACTTTGCTGACGTCCAGCCTGGCCTAATGACAATGGGTGGGGGCAGACTCTCTAACCTGCTGTCCAAGAGGGTCAGGATTTGGTTCTGTGCTAGTAGAACTTGCTCTGCACCTGCGGACTGCAACCCCTGTACCTGTGAATCCGGCGTGTGCGTGGATTGGCTATGA

>Lt098

ATGTGGGTCGTTCTAGTCTGTCTGCTCGGTCTTTGTCTCTTCACTAATGCTGATGACCGCTGCACACTGCAGATATCCACCGGCACCTGTCCAGGCTACTTCCCCCGCTGGTTCTATGACCCGGCCAGTGGTCAGTGCCAGAGCTTCATATACAGCGGATGTAAAGGCAACGCCAACAACTTTCTGTCTGAGGAGGAATGCCACCAGGCTTGCGTCAGTCGTTGA

>Lt099

ATGCCGCGATACTTCTTCCATCAAAGCTCTCAGACCTGCGAGCTGTTTTACTACGGAGGCTGCGGTGGCAACAGCAACAACTTCAGAACTCTGGAAGGCTGTGAAGGTGCCTGCGTCTCCGGTGAGGCTGTGCAGGACGTGTGTGCCCTTCCTAAAGTAGCAGGACCTTGCTTCGCTGCCTTCCCCAGATTTTACTTCGATAAAACAGCAGGCAGGTGCAAGACGTTCACGTATGGAGGGTGCCATGGAAACCAGAACAACTTCCGTTCTCTCAGAGCTTGCAGAAACACATGTCCAGGCAACTGA

>Lt100

ATGGTGAAGTACAACGCCAAAAGGGGAACACCAACTTTATCCAAAAGAACGTCAGATCCCGGGAGATGGTGGGAGAAACAGCTCGGGAGTTGGTCCACGAATTGTGAATGGGACAGCGGTTGTTCTGGCGGTTGTAATGGTCATTACTGCACAACGTGGCGATGA

>Lt101

ATGGTGCGTGTGTTCATTGCGATGTTCTTCCTCTGGGCGCTAACAGAGGGCTGGCCACGTTTGCATGATCGTAACTGTCAGAATGGGCCTAATATGCATCACACATACAGGTGCCGTTCAAGACAGCGATGTGCAATCATTCGTAAAAGGAACGGACAATTGACGTGCGAGCTGAAGTGTAAGTGTGAATCTGTCGGCGACTGTCTTCAGGGTGAAGTCGTCGATTGGGATGTCAGGACTGTCAAAACCTACACCTGCCCGTAG

>Lt102

ATGGTGACCTCAGTTTCAATTGCCTGTCCATGTCTATGGCTTTGGTTTGGTTCTGTTGGTATCACCAGTACAGGCAAATTCATGGGGACTGTCGTTCGGATGGAGGTGCGCCGCATTTGGGGGAGGGAGAGCCCAGGCATGCTGGTTCCATGA

>Lt103

ATGGTGACTTCAGTTTCAATAATCCTTCCACATCTTTGGCCTCAGTTTGATTCTGTTGGGGTCACCAGTGATAGCAACTTCATGGGGGGCTGTCGCTGGATGGAGAGAGAGCTCACTTATACCAACTATGCAACTAAGCAATTATTGTTGTCAGTACAGAGCGTGGTAGGTGGTTTCCTGCATCTGTTGGCTGAGAACTGA

>Lt104

ATGGTGACCTCAGTTTCGATGTCCTCTCCACGTCTGTGGCCTTGGTTTGGTTCCGTCGGGGTCACCGGTATCGGCGAGTCCATGGGGGCTGTCGTTGGACGGACGTGGTGGCGCCCTCCGGAGGGAGAGCTCACTCACGCCGGCTCCGCGACTAAGCAATTATTGTCGTCAGTAGGGGGCTTAGTAGGTGGTGTCCTGCGTATGTTAGATCAGAACAGGCGCCACTGA

>Lt105

ATGACTCTGATGGGATTCGAACCCACGACCTCCCAGCCGGGAGTCCGCGACGCTAACCACTTCGCCACGGCGGCTGGTTTTCTTTTTTTTTTTTTTTTTTTcgGCATTAAAAAAAAAAAATGTCTCATATTCTGTGGCCCTTCATGGCTTGCTCTCATGGTGACCTCAGTGTCAGTGTCCTTTCCACATCCGTGGTCTTGGTTTGGTTCCGTTGGGGTCACCGGTATCGGTGAGTCCATGGGGGCTGTCATTAGATGGACATGGTGGTGCCCTCAGGAGGGAGAGCTCATTCACACTGGCTCCTCGACTAAGCAATTATTGTCATCAACAGAGGGCTTAGTAGGTTGTGTCCTGCATATGTTAGATCACAACAGGCACCACTGA

>Lt106

ATGTGGACCAACAAAGACCTGCCCGCAGTCTTCGCCTTGGTTCTGATCCTGGCCTGGGCCCAGTGCTGCTGGGCAGACACTGACATCTGTAACACGGACGAAGTCGACGATCTGAGTGCCACTGCCATAAGCGGTGACATGTTTTTGGAGCTCGCTAATGATGAAAACTACTTTCTTGCGCAACACTTCGCAAAAGTGTCATATCACTTAACTGCCTTTCAAGAAAGTGGTAGCCAACGATGGAATCTCACGGGGTATTCTTACACCAGCGCTGGTGCATGTTCCGAAAGATCCTTCTTGTGTACAGTGAACGACAACTATTGCAGATACAACGATGGCAAAAAAATATACGTCATCGCTTACGACACCAACAACGTCGTGCTCTACCAACCCAACAATAGTGCTAATACGGTGGACATTTTGGAAATCTATGCCCGATCGCATGGCGCTGCGCAGGTAGAAGTCGACGGCCTTGATGTTGCCATCCGTAATGCAATTCGGAACAAATGCAGCAATGCAGCCACCACCAGCAGTTTCTACAGAAAAAGATTTGGGACCTGGAGCAGCGCAGCAAGCTGCTGA

>Lt107

ATGTCTGTCTGGTCCCCAGCTGGTCTcgGCCGTTCATGTTGCATCAACAAAATGTACCAATGTCTCAAGGGTCATCCTGGCCAGGAACATCTTTACTATACACAATGTCGTTTTGATGCCGTTGCACCTTGTGGAGTTGACGCTTCCCCGAGCTGTTGCAATGCATACATGTTTTGCATTAAGAAGTATGTGCAAGACTATGGCCTTGACGTGAGCCATAACAATTGCAAAGACAGACCGTGTAATCCGCCTGGCCGCTGA

>Lt108

ATGAACATGCGGATGACGATCGTTGTGTTTGTAGTGGTGGCCACAGCAGCCACTCTCGTTGGCTCCACTCCATTGGAAGAACGACAGACCGTGCCCTCCAGTTGCTGCGTCAGGAAGACTGTCGAATGTGCTATGTACACCTGCCCAGAGGTCCCCTTATCTTGCACTGAGGAGTGCTATAAGGAAAAGGCCACTCCCTTATGTGGAGGTGACCCTGGCGATGGCTGTTGCCCTGATTTTATCCGGTGTTACATTAACTGCTTAACTAATGGACCTGGACAACCTTTACCCTGCTATCAAAAGTGCGACAATGGGAGTTGTTTGCGGTAA

>Lt109

ATGAACATGTCGATGACGCTCAGCGTGTTTGTAATGGTTGTCATGGCAGCCACTGTCATTGGTTCCACTCTGTCACAAGAACCAGACCTCAGTCGCATGGAACGCAACAGCCGTTCATGTTGCATCAACAAAACGTACCAGTGTCTGAAGGGTTATCCCGGCCAGGAACATATTTACATCACAAAATGTCACTTTGATGCCGCTGCACCTTGTGGAGCTGACGTTTATCCGGGCTGTTGCAATGGATATATGTATTGCATTGGGATATACGTGCAAGATAATGGCCTTGAACCCACCCATAACTATTGCAAAGACAGACATTGTAATCCGCCTAGCCGCTGA

>Lt110

ATGAACATGTCGATGACGCTCAGCGTGTTTGTAATGGTTGTCATGGCAGCCACTGTCATTGGTTCCACTCTGTCACAAGAACCAGACCTCAGTCGCATGAAACGCTACAGCCGTTCATGTTGCATCAACGAAACGTACGAATGTCTGAAGGGTTATCCTGGCCAGGAACATATTTACTACTCGAAATGTCACTTTGATGCCGCTGCACCTTGTGGAGCTGACGTTTATCCGGGCTGTTGCAATGGATATATGTATTGCATTGCGATATATGTGCAAGACAATGGCCTTGAACCCACCCATAACTATTGCAAAGACAGACCGTGCAATCCGCCTAGCCGCTGA

>Lt111

ATGGTGACCTCAGTGTTGATGTCCTCTCCATGTCTATGGCCTTGGTTTGATTCTGCCAAGGTCACCGGTATCAGCAAGTTTATAGGGACTGTCACTGGATGGATATGCTGGCAAAGGAGAGAGCACACTCATGCCTGCTCTGTGACTGAGCAATTACTGTCATCAGTAGGGGGATTAGTAGGTGGTGTCCTGCATATG

>Lt112

ATGCAACTGTACACGTATCTGTATCTGCTGGTGCCCCTGGTGACCTTCTACCTAATCCTAGGCACGGGCACGCTTGCTCATGGAGACGCACTGACTGAACGCCGTTCGGATGACGCTACAGCGCTGAAACCTGAGCCTGTCCTCCTGCAGAAATCCGCTGCCCGCAGCACCGACGACAATGGCATGGACAGGTTGATTAAGAGGAAGAGGATTCTGAAAAAGCGAGGAAACACGGCCAGAGGCCCCGAAGAAGATTCAGAGACAACGGTTGAGGAACTCCATGAAATAGGAAAAAGATAA

>Lt113

ATGCAGACGGCCTACTGGGTGATGGTGATGATGATGGTGGGGATTACAGCCCCTCTGTCTGAAGGTCGTAAATTGAACGACGCAATTCGGGGTTTGGTGGCAGATTACTTAACCCCACAGCTTTTGCAAAGTCTGGTTTCCGCTCCTTATCCTGAGTTTCAGCTTGACGACCCTAATCTGGAGATACCCGTATGTATCTGGAAGGTATGTCCACCAATCCCATGGAGACGACGTGATCTTAAGAAAAGAAACAAATGA

>Lt114

ATGCAGACGGCCTACTGGGTGATGGTGATGATGATGGTGGGGATTACAGCCCCTCTGTCTGAAGGTCGTAAATTGAACGACGCAATTCGGGGTTTGGTGGCAGATTACTTAACCCCACAGCTTTTGCAAAGTCTGGTTTCCCGTCGTCATCGTGTGTTTCATCTTGACAACACTTATCTGAAGATACCCATATGTGCCTGGAAGGTATGTCCACCAACCCCATGGAGACGACGTGATCTTAAGAAAAGAAACAAATGA

>Lt115

ATGCAGACGGCCTACTGGGTGATGGTGATGATGATGGTGGGGATTACAGCCCCTCTGTCTGAAGGTCGTAAATTGAACGACGCAATTCGGGGTTTGGTGCCAGATGACTTAACCCCACAGCTTTTGCGAAGTCCGGTTTCGACTCCTTATCCTGAGTTTCATCTTGATGAACCTTATCTGAAGATACCCGTATGTATCTGGAAGATATGTCCACCAAACCTATTGAGACGACGTGATCTTAAGAAAAGAAACAAAGTACGTCAGACAACCGCCACAACTTGA

>Lt116

ATGCCTGTGGATTTAGAGATCCTGAAAGCTCCGACTAAGGAATCTCGTAAAGATTTTGAGATGAGAATTGAACTCCTACGTTCAAAGAGACAGTGCTGTAGGCCTGCTAACATGTCTTGCTGTCAGGGGTAA

>Lt117

ATGAACGCCTGTGCCACAGCCAACAGCTACATGGACAACCCATGGTCTCTGATGAACATTGCGGACTTTAAAGAAAGGAGTACATACTATTCAATGTACACCGAAATGGTAACCTGCCTGAACTATTTTCTCCAGAGAAGACTATGA

>Lt118

ATGCAGAGAGGGGCCGTGCTGCTCGGGGTGGTCGCCTTCCTTGCCCTGTGGCCCCAGGCCGCTGCCAGGGTGTACGATTTGAACGAGCCTGACGTGTGGGGCATGGTCGACTATGCACAGAGAGCGATGCATTCCTGTGCCATAGTGAATGACTACGACGACAGGCGTTGGTCGAGTTACAACGTTGCGGAATTTAAAGACAGGAGTCTATTCCGAACAATGGTGACCGACTTGCAAGGCTGCCTGAACTATTTTTTCCAGATAAGACCATGA

>Lt119

ATGAACGGCATGTACCTGGGGTATGGTTATCAGAACGGTCTTGTCTCGCCATACATGAACGGCATGTACCTGGGGTATGGTTATCAGAACGGTCTTGTCTCGCCATACATGAACGGCATGTACCTGGGGTATGGTTATCAGAACGGTCTTGTCTCGCCATACATGAACGGCATGTTCCCGGGTTATGGCTACCCCAACAGTCTCCTCTTCACCTCCTCCATGCAAGCTTTACCCAGGTACCTCCTCCCTGCCAATGGCACCTATCCAGGGGGCTTCTTCCCCGGCGGCTTTGCTGGAGCTTATCCCATGTACCCGTATTCGCCTGGCTACCTCGCTAATCCCATGATGTATTCCGGGTACGCCTTTGCTGGTGGCTATCCTGGTTACCCCATGCCAGGTATGTTTCCTGGGTACCCCTATCAAGCCGGGTATCCCGGGCTCGGTATGCAGCCCTGGTACGGCGGTATGGGCTTGATGGCCTTCCGGCCTCCTCTAGGGGGCATGGGTTCGCTCTTCTATCCTCAGCCGGGGTACGTCGGGTGGAGCTACAGCCACTGGCACCAAACCAATGCGTCGAAGGAGTCTCCTCTTCTCAAGGCGATCTACAACCTGGGTGGCGAGTCCACAGCACCACAAAGACCCTCACAGGAGGAAATCAAGAAGCTGCTGTGTGACCATCCCTGGGTGAAAATGATGCTTTTCAATAACACCGACGACGAAGTTGTTAAAGCGCTGAGAGAGGCCAAATCCTTGGATGAGCTGAAAAAGAAGCTGAACCTGAGTGACGATAAGGTGACCAACCTACGCACGGTCTTCAAGGCGGTGAAAGAGGACAGCGTCAAGCCAATCATAGAGCTGGGGGAGAAGGAGAATCACTGGGACGACTTCAAGAAGCCCTTCAAGGCCATCCTGGACAGTGGATTCCAGGTCTAG

>Lt120

ATGACGTCTGTTCAGTCTGTGACCTGCTGCTGCCTGCTGTGGCTGATGCTCTCTCTCACTCTCGTCACTCCTGGTTCCCCTGGACCTGCACAGCTGCCTGGGCATCGCGCTGCTAGAGTTCCTGCAGAGCCGATATTGGAAGAGATATGCCCTGACATGTGCAACAGTGGAGAAGGGGAGATCTTTTGCACCTGCGGATCAAGACAGTTCGTGGTTACTCTACCGGTCATAGAGCGAAAAAGATCGATGGCGGTGTGA

>Lt121

ATGACTTGGGCATCCATCTCCTCTGCGCATCTGTGCCCTTGGACAATTTGTGTTGGGGTCTCTGGTATAGGGTCTGTTGTTGGAGAGACATGGTGGCACCCTTTAGTGTATGCTGGATCAGAACAGCCACCACTGAACATCAACGAAGTGACGTTGCAGCAGCTCAGTGTCTCTCCTCTGATGGGATGCCTCGTAACAACCTGA

>Lt122

ATGGGGGCGGATGTTGGACGGATATGGTGGCACCCTCCAGAGGAAGAGCTCACTCATGCAGGCTCCACGACTATGCAGTTGTCGACAGTAGTAGTAGTAGGGCTTTGCAGGTGTTTCACTGTGTATGTTGGACCAGAACAGGCACCACTGAACTCTACCAAAGTGACTTGGCAGCAGTACAGTGTCTCCTCTGcgCCGGCATGA

>Lt123

ATGCCCGAGGAGAAGAAGTTGTTGTCAGACAGTGCGACAACCATGTCAGACCGTTCTGTCATAGGCCAGAGTACAGAGTTCACGTCCATGTTGACGGATGGCCAGACTCTGCCAGTGTCCcgCTCCACGTCTATGGCCTTTGTTTGCCTCCGTCGGGGTCACGGTATCTGCAAGTTCATAGAGGCTGTCATTGGACAGACATGGTGGTGCCCTCCGGGGGAGCTCACTCGTGCAGGCTCCATGACTAAGCAGTTATTGTCATCTGTAGGGGGTTTAGTAGTTGGTGTCCAACATACACCAATTTGA

>Lt124

ATGCATACACTCGGTAGAACGTCACGTCTGGAGGCACTAGCCCCAGGTGCAAGCATAAAGATGGCCAGGATTTTTTTTTTTTTTTTTTcgTCCAGCGGCGACCTTGGAGCTCCCATCCCCCATCTACAGTCCCAAGAAAGGAGGTGCCTCCGGGGGAGCGTCTGCATGTGTTTTGATGCCCGAGGAGAAGAAGTTGTTGTCAGACAGTGCGACAACCATGTCAGACCGTTCTGTCATAGGCCAGAGTACAGAGTTCACGTCCATGTTGACGGATGGCCAGACTCTGCCAGTGTCCcgCTCCACGTCTATGGCCTTTGTTTGCCTCCGTCGGGGTCACGGTATCTGCAAGTTCATAGAGGCTGTCATTGGACAGACATGGTGGTGCCCTCCGGGGGAGCTCACTCGTGCAGGCTCCATGACTAA

>Lt125

ATGCAGACGCTCCCCCGGAGGCACCTCCTTTCTTGGGACTGTAGATGGGGGATGGGAGCTCCAAGGTCGCCGCTGGAcgAAAAAAAAAAAAAAAAAATCCTGGCCATCTTTATGCTTGCACCTGGGGCTAGTGCCTCCAGACGTGACGTTCTACCGAGTGTATGCATACTACCAGCGGTAACGGGACCGTGCAAAGCGCGTTGGCTGAAATATTACTTCGATATGGCTTCTTACGACTGTCGAATTTTCATTTACGGAGGATGTGGTGGCAACGGCAAC

>Lt126

ATGGTGACCTCAATTTCAATGTCCTCTCCATGTCTGTGGCCTGGGTTTGGTTCCATCGGGGTCACTGATATTGGCGAGTCCATGGAGGCTGTCACTGGACAGACGTGGTGGTGCCCTCCAGAGGGAGAGCTCACTCACACCGGCTCCACGACTAAGCCATTATTGTCAACAGTAGGGGGCATAGTAGGTGGTGTCCTGAGTGTGTTGGATCAGAACAAACACCAT

>Lt127

ATGAAGGTGCTGGAATCAGCCTTGTGGACACTGGCAGCTCTGGCACTACCACGGATTGCGGCTCAGGATTCCAGTAGGGCTGAGTTATGCAAGATCAACAGCAACGGTTGCAGTGTTCCTTTTCCGGATGTACCATGCCAAGAGCATTTCCGTCCTGCTTGTGACATTCACGATAACTGTTATCTTTGTGGAGCGCTTTTCGGCTTCACGCAGGGCAAATGTGACAGGGCTTTTCTCACCCACATGACTGCTCTGTGTGCCCGGGGTACTGATGACGAAGGCTTCTGCCTAGAGAAACGGAAACGAAGAGAAGCTTCCAGCATGTCCATCACCACCCCTCTCAGACAACTACGGCTTCTGGAGAAACTCCTGCCCCCGAATTCCTTGTCGGATCGTGACCCACGTCAGCCCCATCCACGTTCTGCCACCTGCACTGAATGGGCTTTGATGTATTTTGATGCGGTCCAGGCCATGGGCTGGTTGTACTTCTACAACACGGCCAATACCACGTACTGCCCCAGGTTTAAGACTTGTATGCCTGAGGTTTGA

>Lt128

ATGGTGATGTCAATGGTGGCGGCATCAGGAACTGAAAACCACGCGATGTGCAAGGTCCACAGCGACGGTTGTAGCGTACCCTTAGGTCTGCCCTTCTTCTACAAGTCCAGATTCACACCTGCCTGCCATATCCACGACGTCTGCTACGGATGTGGCGCAAAGTACGGACGGACTAAAGAGGACTGCGACATCGCCTTCCTGCAGGACATGATCCACGCCTGCCATACGGCCAGACGGCGCCGATCCTCCAGGAACAAACGCACTCTCTGCGTCAGCGTGGCCACAAACATGTATTACAAGGCCGTGCACTGGTTTGCCAACCCTCATTATCATACTGCGGACGACTTAGACCCTCATTGCCATGAGAACTGGGTGCCAAAGTGTCTGCCTCCAAAGTACCATTAA
