# Supplementary material for: Diversity of Conopeptides and Their Precursor Genes of Conus Litteratus
Source: Mar Drugs. 2020 Sep 14;18(9):464. doi: 10.3390/md18090464 (PMC7551347; doi:10.3390/md18090464)
Supplement: Supplementary file 1 [file marinedrugs-18-00464-s001.zip › Supplementary/S8-B1 and J superfamily gene.docx]

B1 superfamily conotoxin genes

>Lt-B1-01

ATGCAACTGTACACGTATCTGTATCTGCTGGTGCCCCTGGTGACCTTCTACCTAATCCCAGGCACGGGCACGCTTGCTCATGGAGACGCACTGACTGAACGCCGTTCGGATGACGCTACAGCGCTGAAACCTGAGCCTGTCCTCCTGCAGAAATCCGCTGCCCGCAGCACCAACGACAATGGCATGGACAGGTTGACTCAGAGGAAGAGGATTCTGAAAAAGCGAGACAACACGGCCAGAGGCCCCGAAGAAGATTCAGAGACAACGGTTGAGGAACTCCATGAAATAGGAAAAAGATAA

>Lt-B1-02

ATGCAACTGTACACGTATCTGTATCTGCTGGTGCCCCTGGTGACCTTCTACCTAATCCTAGGCACGGGCACGCTTGCTCATGGAGACGCACTGACTGAACGCCGTTCGGATGACGCTACAGCGCTGAAACCTGAGCCTGTCCTCCTGCAGAAATCCGCTGCCCGCAGCACCGACGACAATGGCATGGACAGGTTGATTAAGAGGAAGAGGATTCTGAAAATGCGAGGAAACACGGCCAGAGGCCCCGAAGAAGATTCAGAGACAACGGTTGAGGAACTCCATGAAATAGG

>Lt-B1-03

ATGCAACTGTACACGTATCTGTATCTGCTGGTGCCCCTGGTGACCTTCTACCTAATCCCAGGCACGGGCACGCTTGCTCATGGAGACGCACTGACTGAACGCCGTTCGGATGACGCTACAGCGCTGAAACCTGAGCCTGTCCTCCTGCAGAAATCCGCTGCCCGCAGCACCAACGACAATGGCATGGACAGGTTGATTAAGAGGAAGAGGATTCTGAAAAAGCGAGGAAACACGGCCAGAGGCCTCGAAGAAGATTTAGAGACAGCGGAGATGGTTGAGGAACTCCATGAAATAGGAAAAAGATAA

J superfamily conotoxin genes

>Lt-J-01

ATGACGTCTGTTCAGTCTGTGACCTGCTGCTGCCTGCTGTGGCTGATGCTCTCTCTCACTCTCGTCACTCCTGGTTCCCCTGGACCTGCACAGCTGCCTGGGCATCGCGCTGCTAGAGTTCCTGCAGAGCCGATATTGGAAGAGATATGCCCTGACATGTGCAACAGTGGAGAAGGGGAGATCTTTTGCACCTGCGGATCAAGACAGTTCGTGGTTACTCTACCGGTCATAGAGCGAAAAAGATCGATGGCGGTGTGA

>Lt-J-02

ATGACGTCTGTTCAGTCTGTGACCTGCTGCTGCCTGCTGTGGCTGATGCTCTCTCTCACTCTCGTCACTCCTGGCTCTCCTGGACCTGCACAGCTGCCTGGGCATCGCGCTGCTAGAGCTCTTGCAGAGCCGATATTGGAAGAGATATGCCCTGACATGTGCAACAGTGGAGAAGGGGAGATCTTTTGCACCTGCGGATCAAGACAGTTCGTGGTTACTCTACCGGTCATAGAGCGAAAAAGATCGATGGCGGTGTGA

>Lt-J-03

ATGACGTCTGTTCAGTCTGTGACCTGCTGCTGCCTGCTGTGGCTGATGCTCTCTCTCACTCTCGTCACTCCTGGTTCCCCTGGACCTGCACAGCTGCCTGGGCATCGCGCTGCTAGAGTTCCTGCAGAGCAGATGATGGAAGAGCTATGCCCTGACATGTGCAACAGGGGAGAAGGGGAGATCATTTGCACCTGCGTATTAAGACGGCACGTGGTTTCTCCATCGATCAGAGAGCGAAAAAGATCGATGGCGGTGTGA

>Lt-J-04

ATGACGTCTGTTCAGTCTGTGACCTGCTGCTGCCTGCTGTGGCTGATGCTCTCTCTCACTCTCGTCACTCCTGGATCCCCTGGACCTGCACAGCTGCCTGGGCATCGCGCTGCTAGAGTTCCTGCAGAGCCGATATTGGAAGAGCCATGCCCTGACATGTGCAACAGTGGAGAAGGGGAGATCTTTTGCACCTGCGGATCAAGACAGTTCGTGGTTACTCTACCGGTCATAGAGCGAAAAAGATCGATGGCGGTGTGA

>Lt-J-05

ATGACGTCTGTTCAGTCTGTGACCTGCTGCTGCTGCCTGCTGTGGCTGATGCTCTCTCTCACTCTCGTCACTCCTGGATCCTCTGGACCTGCACAGCGGCCTGGGCATCGCGCTGCCAGAGTTCCTGCAGAGCCGATATTGGAACAGCTATGCCCTGAAATGTGCAACAGGGGAGAACTGGAGTTCTTTTGCACCTGCGGATCAAGACAGTTCGTGGTTACTCTACCGGTCATAGAGCGAAAAAGATCGATGGCGGTGTGA

> Lt-J-06

ATGACGTCTGTTCAGTCTGTGACCTGCTGCTGCCTGCTGTGGCTGATGCTCTCTCTCACTCTCGTCACTCCTGGTTCCCCTGGACCTGCACAGCTGCCTGGGCATCGCGCTGCTAGAGTTCCTGCAGAGCCGATATTGGAACAGCTATGCCCTGACATGTGCAACAGGGGAGAACTGGAGATCTTTTGCACCTGCGGATCAAGACAGTTCGTGGTTACTCTACCGGTCATAGAGCGAAAAAGATCGATGGCGGTGTGA

> Lt-J-07

ATGACGTCTGTTCAGTCTGTGACCTGCTGCTGCCTGCTGTGGCTGATGCTGTCTCTCACTCTCGTCACTCCTGGATCCCCTGGATCTGCACAGCTGCCTGGGCATCGCGCTGCTAGAGTTCCTGCAGAGCAGATGATGGAAGAGCTATGCCCTGACATGTGCAACAGGGGAGAAGGGGAGATCATTTGCACCTGCGTATTAAGACGGCACGTGGTTTCTCCATCGATCAGAGAGCGAAAAAGATCGATGGCGGTGTGA

> Lt-J-08

ATGACGTCTGTTCAGTCTGTGACCTGCTGCTGCCTGCTGTGGCTGATGCTGTCTCTCACTCTCGTCACTCCTGGATCCCCTGGATCTGCACAGCTGCCTGGGCATCGCGCTGCTAGAGTTCCTGCAGAGCAGATGATGGAAGAGCTATGCCCTGACATGTGCAACAGGGGAGAAGGGGAGATCATTTGCACCTGCGTATTAAGACGGCACGCGGTTTCTCCATCGATCAGAGAGCGAAAAAGATCGATGGCGGTGTGA
