# Supplementary material for: Diversity of Conopeptides and Their Precursor Genes of Conus Litteratus
Source: Mar Drugs. 2020 Sep 14;18(9):464. doi: 10.3390/md18090464 (PMC7551347; doi:10.3390/md18090464)
Supplement: Supplementary file 1 [file marinedrugs-18-00464-s001.zip › Supplementary/S9-F superfamily gene structure.docx]

**The gene structure of F superfamily**

The blue bold italic is the signal peptide sequences, the blue regular font is the precursor peptide region, the red bold is mature peptide sequence, the black capital letter is the exon region, the black lowercase italic is the intron region, and the green shaded region indicates simple repetition sequences

**LtF101**

***M  M  Q  R  G  A  V  L  L  G  V  V  A  F  L  G  L  L  P  Q*** 
ATGATGCAGAGAGGGGCCGTGCTGCTCGGGGTGGTCGCCTTCCTTGGCCTGTTGCCCCAG
***A  A  A***  K  V  Y  N  L  H  E  T  E  V  W  A  V  V  A  Y  S  
GCCGCTGCCAAGGTGTACAATTTGCACGAAACTGAAGTGTGGGCCGTGGTCGCCTATTCA
K  R  **L  M  N  A  C  A  I  A  N  S  H  M  D  N**              
AAGAGACTGATGAACGCCTGTGCCATAGCCAATAGCCACATGGACAA*gtaagagagagag
agagagagagagagagagagagagagagagagagagagactttgacactgatattttatt
gtcattacgaaagaggtctcttgacaaggggagggaggtaacagaacaataacatttccc
tcttcctttcctgcaaacaaatagtacactttcattcctagtgtgtatcaaatcaaaggt
taacaaaaacaaataaattcgtaacatttccttatgtccccacacacaggagtaatacag
taaggtaaaaaaaaaaaaggaaaagaaaaagaaacgaaaaaaaaaaatcgaaaaaaaaac
cgcttaagactgactcgaccatccacccaattaaatgatgacgttgtagcttactaatca
cttccacatgcgatgaatgataatgttcattcatatatctcttataatgtattatcttaa
atttcactatccttcatctgtctaattttcaatgcttccgtgatgaatttcgcaattgat
ttaattacatgttcatcatctgatgataacactttggcgacattttgtcttttcgttata
tctaacaggaaaaatgaacaattttttttttgttttttttagagagagagagagagagag
agagagagagagagagagagagagaggcgaaaagttgaatagtttcaacgtgaggtgact
gtaggcggaaattgaaacaaattttaaaagtaattttaaggtaactccaccggtttacat
ggacaaagaaataaccagacaaaacatgacgcgtttcgacctcttaggtcttcttcaggc
aagataacattcaggacaatgaaaacgcaggaacaagcaatcaattgtcttcttgaattc
ggcgcaagatgagcattgatggttcatgttaaccatgccaagtcatttactttttttttt
taagaaaagaaaagttttatcacaagacacgctcttataaacctgctcacaaagcggagg
aaactagggggtttgaggggaacaaaggggtgaggaagttggggggaagaggagggaggg
gggcaaagaccgacaaagggaaggggctataatattcaccctctctctctctctctctct
ctctctctctgtaaacatgtacacttgcgtgaacgtgtgcatacctgcatacgtgcatga
gtgtgtgtgtgtatgtgtgtgtgtgtgtgtgtgtgtgtgcgtgtgcgtgtgtgtgtgtgt
gtgtgtgcacacgcgcacatccgcttgtatgtgtgtttgtgtgtgtgacatatctttcag* **P  W  S  V  V  D  V  G  D  F  E  E  R  S  L  Y  N  S  L  Y**
CCCATGGTCTGTGGTGGATGTTGGGGACTTTGAAGAAAGGAGTCTATACAATTCATTGTA
 **K  A  M  V  S  C  L  E  D  F  F  Q  Q  R  P** *  
CAAGGCAATGGTATCCTGCCTGGAAGATTTTTTCCAGCAAAGACCATGA

Intron:1273bp

**LtF102**

***G  A  V  L  L  G  V  V  A  F  L  A  L  W  P  Q  A  A  A***  K  
GGGGCCGTGCTGCTCGGAGTGGTTGCCTTCCTTGCCCTGTGGCCCCAGGCCGCTGCCAAG
V  Y  D  L  H  E  T  E  V  W  A  V  V  A  Y  S  K  R  **L  M**  
GTGTACGATTTGCACGAAACTGAAGTGTGGGCCGTGGTCGCCTATTCAAAGAGACTGATG
**N  A  C  A  L  A  N  G  H  M  D  N**                          
AACGCCTGTGCCCTAGCCAATGGCCACATGGACAA*gtaagagagagagagagagagagag
agagagagagagagagagagagagagagagagactttgacactgatattttattgtcatt
acgaaagaggtctcttgacgaggggagggaggtaacagaacaataactttccctcttcct
ttcctgcaaacaaatagtacactttcattcctagtgtgtatcaaatcaaaggttaacaaa
aacaaataaattcgtaacatttccttatgtccccacgcacaggagtaatacagtaaggta
aaaaaaaatgaaaagaaaaagaaacgaaaaaaaaatcgaaaaaaaacctcttaagactga
ctcgaccatccacccaattaaatgatgacattgtatcttactaatcacttccacatgcaa
tgaatgataatgttcattcatatatctcttataatgtattatcttaaatttcactatcct
tcatctgtctaattttcaatgcttccatgatgaatttcgcaatcgatttaattacatgtt
catcatctgatgataacacttcggcgacattttgtctttttgttatatctaactggaaaa
atgaacaattcttgtttttttttttttgtttgtttgtttgtttgtttgtttttgagagag
agagagagagagagagagagagagagagagagagagagagagagagagagagaggtgaaa
agttgagtagtttcaacgtgaagtgactgtaggcggaaattgaaacaaattttaaaagta
attttaaggtaactccaccggtttacatggacaaagaaataaccagacaaaacatgatgc
gtttcgacctcttaggtcttcttcagtcaagataacattcaggacaatgaaaacgcagga
acgagcaatcagttgtcttctcgaattcgacgcaagatgagcatagatggttcatgttaa
ccatgccaagtcatttacatttttttttaagaaaagaaaagttttatcgcaagacacgct
cttataaacctgctcacaaagcagaggaaactagggggtttgcggggaacaaaggggtga
ggaagttgggggggaagaggagggaggggggcaaagactgtcaaagggaaggggctataa
tattcaccctctctctctctctctctctctctctctctctctctctctctctctctgtgt
aaacatgcacacttgcgtgcacgtgtgcatacctgcatgcgtgcatgcatgtgtgtgtgt
atgtatgtgtgtgtgtgtgtgtgtgtgtgtgtgtgtgtgtgcacgcgcacatccgcttgt*                                **P  W  S  V  V  D  V  K  D**  
*atatgtgtttgtgtgtgtgacatacctttcag*CCCATGGTCTGTGGTGGATGTTAAGGAC
**F  E  E  R  S  L  F  H  S  M  Y  K  A  M  V  H  C  L  E  D**  
TTTGAAGAAAGGAGTCTATTCCATTCAATGTACAAGGCAATGGTACACTGCCTGGAAGAT
**F  F  Q  Q  R  P**  *  
TTTTTCCAGCAAAGACCATGA

Intron: 1317bp

**LtF103**

***M  M  Q  R  G  A  V  L  L  G  V  V  A  F  L  G  L  L  P  Q*** 
ATGATGCAGAGAGGGGCCGTGCTGCTCGGGGTGGTCGCCTTCCTTGGCCTGTTGCCCCAG
***A  A  A***  K  V  Y  N  L  H  E  T  E  V  W  A  V  V  A  Y  S  
GCCGCTGCCAAGGTGTACAATTTGCACGAAACTGAAGTGTGGGCCGTGGTCGCCTATTCA
K  R  **L  M  N  A  C  A  I  A  N  S  H  M  D  N**              
AAGAGACTGATGAACGCCTGTGCCATAGCCAATAGCCACATGGACAA*gtaagagagagag
agagagagagagagagagagagagagagagagagagagagagagactttgacactgatat
tttattgccattacgaaagaggtctcttgacaaggggagggaggtaacagaacaataaca
tttccctcttcctttcctgcaaacaaatagtacactttcattcctagtgtgtatcaaatc
aaaggttaacaaaaacaaataaattcgtaacatttccttatgtccccacacacaggagta
atacagtaaggtaaaaaaaaaaaaggaaaagaaaaagaaacgaaaaaaaaaaatcgaaaa
aaaaaccgcttaagactgactcgaccatccacccaattaaatgatgacattgtagcttac
taatcacttccacatgcgatgaatgataatgttcattcatatatctcttataatgtatta
tcttaaatttcactatccttcatctgtctaattttcaatgcttccgtgatgaatttcgca
attgatttaattacatgttcatcatctgatgataacactttggcgacattttgtcttttc
gttatatctaacaggaaaaatgaacaatttttttttggtttttttgagagagagagagag
agagagagagagagagagagagagagagagagagaggcgaaaagttgagtagtttcaacg
tgaggtgactgtaggcggaaattgaaacaaattttaaaagtaattttaaggtgactccac
cggtttacatggacaaagaaataaccagacaaaacatgatgcgtttcgacctcttaggtc
ttcttcaggcaagataacattcaggacaatgaaaacgcaggaacgagcaatcaattgtct
tctcgaattcggcgcaagatgagcattgatggttcatgttaaccatgccaagtcatttac
atttttttaagaaaagaaaagttttatcgcaagacacgctcttataaacctgctcacaaa
gtggaggaaactagggggtttgcagggaacaaaggggtgaggaagttggggggaagagga
gggaggggggcaaagaccgacaaagggaaggggctataatattcaccctctctctctctc
tctctctctctctctctgtaaacatgtacacttgcgtccacatgcgcatacctacatgcg
tgcttgagtgtgtgtgtatgtgtgtgtgtgtgtgtgtgtgtgtgtgtgtgtgtgtgtggt
tgtgtgtgtgtgtgtgtgtgtgtgtgtgtgtgtgtgtgtgtgtgtgtgtgtgtgtgtgca*                                                  **P  W  S  V**
*cgcgcacatccgcttgtatgtgtgtttgtgtgtgtgacatatctttcag*CCCATGGTCTG
 **V  D  V  K  D  F  E  E  R  S  L  Y  H  S  L  Y  K  A  M  V**
TGGTGGATGTTAAGGACTTTGAAGAAAGGAGTCTATACCATTCATTGTACAAAGCAATGG
 **S  C  L  E  D  F  F  Q  Q  R  P***  
TATCCTGCCTGGAAGATTTTTTCCAGCAAAGACCATGA

Intron:1322bp

**LtF104**

***G  A  V  L  L  G  V  V  A  F  L  A  L  W  P  Q  A  A  A***  K  
GGGGCCGTGCTGCTCGGGGTGGTCGCCTTCCTTGCCCTGTGGCCCCAGGCCGCTGCCAAG
V  Y  N  L  H  D  T  E  V  W  A  V  V  A  Y  S  K  R  **V  M**
GTGTACAATTTGCACGATACTGAAGTGTGGGCCGTGGTCGCCTATTCAAAGAGAGTGATG
**H  A  C  A  I  A  N  S  H  M  D  D**                          
CACGCCTGTGCCATAGCCAATAGCCACATGGACGA*gtaagagagagagagagagagagag
agagagagagagagagagagagactttgacactgatattttattgtcattacgaaagagg
tctcttgacaaggggagggaggtaacagaacaataacatttccctcttcctttcctgcaa
acaaaaagtacactttcattcctagtgtgtatcaaatcaaaggttaacaaaaacaaataa
attcgtaacatttccttatgtccccacgcacaggagtaatacagtaaggtaaaaaaaaaa
aaagaaaagaaaaagaaacgaaaaaaaaatcgaaaaaaaaacccttaagactgactcgac
catccacccaattaaatgatgacattgtatcttattaatcacttccacatgcaatgaatg
ataatgttcattcatatatcccttataatgtattatcttaaatttcactgtccttcatct
gtctaattttcaatgcttccatgatgaatttcgcaatcgatttaattacatgttcatcat
ctgatgataacacttcggcgacattttgtcttttcgttatatctaactggaaaaatgaac
aattcttgctttttttttttttttttttttttttcttttttttgagagagagagagagag
agagagagagagagagagagcgagagagagagagagaggcgaaaagttgagtagtttcaa
cgtgaggtgactgtaggcggaaattgaaacaaattctaaaagtaattttaaggtaactcc
accggtttacatggacaaagaaataaccagacagaacatgatgcgtttcgacctcttagg
tcttcttcaggcaagataacattcaggacaatgaaaacgcaggaacgagcaatcagttgt
cttctcgaattcgacgcaagatgagcatagatggttcatgttaaccatgccaagtcattt
acattttttttttaagaaaagaaaagttttatcgcaagacacgctcttataaacctgctc
acaaagcggaggaaactagggggtttgcggggaacaaaggggtgaggaagttggggggaa
gaggagggaggggggcaaagactgacaaagggaaggggctataatattcaccctctctct
ctctctctctgtaaacatgtacacttgcgtgcacgtgtgcatacctgcatgcgtgcatgc
gtgtgtgtgtgtatgtatgtatgtgtgtgtgtgtgtgtgtgtgtgtgtgtgtgtgtgtgt
gtgtgtggttgtgtgtgtgtgtgtgtgtgtgtgtgtgtgtgtgtgtgtgtgtgtgtgtgt*                                                     **P  W  L**
*acacgcgcacatccgcttgtacatgtgtttgtgtgtgtgacatacctttcag*CCCATGGC
  **V  V  D  V  K  D  F  E  E  R  S  L  F  H  S  M  Y  K  A  M**TTGTGGTGGATGTTAAGGACTTTGAAGAAAGGAGTCTATTCCATTCAATGTACAAGGCAA
  **V  S  C  L  E  D  F  F  Q  Q  R  P** *  
TGGTATCCTGCCTGGAAGATTTTTTCCAGCAAAGACCATGA

Intron:1337 bp

**LtF105**

***M  M  Q  R  G  A  V  L  L  G  V  V  A  F  L  G  L  L  P  Q***
ATGATGCAGAGAGGGGCCGTGCTGCTCGGGGTGGTCGCCTTCCTTGGCCTGTTGCCCCAG
***A  A  A*** K  V  Y  N  L  H  E  T  E  V  W  A  V  V  A  Y  S  
GCCGCTGCCAAGGTGTACAATTTGCACGAAACTGAAGTGTGGGCCGTGGTCGCCTATTCA
K  R  **L  M  N  A  C  A  I  A  N  S  H  M  D  N**             
AAGAGACTGATGAACGCCTGTGCCATAGCCAATAGCCACATGGACAA*gtaagagagagag
agagagagagagagagagagagagagagagagagagagagagactttgacactgatattt
tattgtcattacgaaagaggtctcttgacaaggggagggaggtaacagaacaataacatt
tccctcttcctttcctgcaaacaaatagtacactttcattcctagtgtgtatcaaatcaa
aggttaacaaaaacaaataaattcgtaacatttccttatgtccccacacacaggagtaat
acagtaaggtaaaaaaaaaaaggaaaagaaaaagaaacgaaaaaaaaaatcgaaaaaaaa
accgcttaagactgactcgaccatccacccaattaaatgatgacattgtagcttactaat
cacttccacatgcgatgaatgataatgttcattcatatatctcttataatgtattatctt
aaatttcactatccttcatctgtctaattttcaatgcttccgtgatgaatttcgcaattg
atttaattacatgttcatcatctgatgataacactttggcgacattttgtcttttcgtta
tatctaacaggaaaaatgaacaattttttttttgtttttttgagagagagagagagagag
agagagagagagagagagagagagagagaggcgaaaagttgaatagtttcaacgtgaggt
gactgtaggcggaaattgaaacaaattttaaaagtaattttaaggtaactccaccggttt
acatggacaaagaaataaccagacaaaacatgatgcgtttcgacctcttaggtcttcttc
aggcaagataacattcaggacaatgaaaacgcaggaacgagcaatcaattgtcttctcga
attcggcgcaagatgagcattgatggttcatgttaaccatgccaagtcatttacattttt
ttaagaaaagaaaagttttatcgcaagacacgctcttataaacctgctcacaaagtggag
gaaactagggggtttgcagggaacaaaggggtgaggaagttggggggaagaggagggagg
ggggcaaagaccgacaaagggaaggggctataatattcaccctctctctctctctctctc
tctctctctcgctctgtaaacatgtacacttgcgtccacatgcgcatacctgtatgcgtg
cttgagtgtgtgtgtatgtgtgtgtgtgtgtgtgtgtgtgtgtgtgtgtggttgtgtgtg
tgtgtgtgtgtgtgtgtgtgtgtgtgtgtgtgtgtgtgtgtgtgtgtgtgtgtgcacacg*                                                 **P  W  S  V**
*cgcacatccgcttgtatgtgtgtttgtgtgtgtgacatatctttcag*CCCATGGTCTGTG
**V  D  V  K  D  F  E  E  R  S  L  Y  H  S  L  Y  K  A  T  V**
GTGGATGTTAAGGACTTTGAAGAAAGGAGTCTATACCATTCATTGTACAAAGCAACGGTA
**S  C  L  E  D  F  F  Q  Q  R  P***  
TCCTGCCTGGAAGATTTTTTCCAGCAAAGACCATGA

Intron:1320bp

**LtF106**

***M  M  Q  R  G  A  V  L  L  G  V  V  A  F  L  A  L  W  P  Q***
ATGATGCAGAGAGGGGCCGTGCTGCTCGGGGTGGTCGCCTTCCTTGCCCTGTGGCCCCAG
***A  A  A*** K  V  Y  N  L  H  D  T  E  V  W  A  V  V  A  Y  S  
GCCGCTGCCAAGGTGTACAATTTGCACGATACTGAAGTGTGGGCCGTGGTCGCCTATTCA
K  R  **V  M  H  A  C  A  I  A  N  S  H  M  D  D**             
AAGAGAGTGATGCACGCCTGTGCCATAGCCAATAGCCACATGGACGA*gtaagagagagag
agagagagagagagagagagagagagagagagagagagagactttgacactgatatttta
ttgtcattacgaaagaggtctcttgacaaggggagggaggtaacagaacaataacatttc
cctcttcctttcctgcaaacaaaaagtacactttcattcctagtgtgtatcaaatcaaag
gttaacaaaaacaaataaattcgtaacatttccttatgtccccacgcacaggagtaatac
agtaaggtaaaaaaaaaaaaagaaaagaaaaagaaacgaaaaaaaaatcgaaaaaaaaac
ccttaagactgactcgaccatccacccaattaaatgatgacattgtatcttattaatcac
ttccacatgcaatgaatgataatgttcattcatatatctcttataatgtattatcttaaa
tttcactgtccttcatctgtctaattttcaatgcttccatgatgaatttcgcaatcgatt
taattacatgttcatcatctgatgataacacttcggcgacattttgtcttttcgttatat
ctaactggaaaaatgaacaattcttgtttttttttttttttttttttttttttttttttt
gagagagagagagagagagagagagagagagggagagagagagagagagaggcgaaaagt
tgagtagtttcaacgtgaggggactggagggggaaattgaaacaaattttaaaagtaatt
ttaagggaactcccccggtttacatggacaaagaaataaccagacaaaacatgatgcgtt
tcgacctcttaggtcttcttcaggcaagataacattcaggacaatgaaaacgcaggaacg
agcaatcagttgtcttcccgaattcgacgcaagatgagcatagatggttcatgttaacca
tgccaagtcatttacattttttttttaagaaaagaaaagttttatcgcaagacacgctct
tataaacctgctcacaaagcggaggaaactagggggtttgcggggaacaaaggggtgagg
aagttggggggaagaggagggaggggggcaaagactgacaaagggaaggggctataatat
tcaccctctctctctctctctgtaaacatgtacacttgcgtgcacgtgtgcatacctgca
tgcgtgcatgcgtgtgtgtgtgtatgtatgtatgtgtgtgtgtgtgtgtgtgtgtgtgtg
tgtgtgtgtgtgtgtggttgtgtgtgtgtgtgtgtgtgtgtgtgtgtgtgtgtgtgtgtg
tgtgtgtgtgtacacgcgcacatccgcttgtacatgtgtttgtgtgtgtgacataccttt* **P  W  L  V  V  D  V  K  D  F  E  E  R  S  L  F  H  S  M**
*cag*CCCATGGCTTGTGGTGGATGTTAAGGACTTTGAAGAAAGGAGTCTATTCCATTCAAT
 **Y  K  A  M  V  S  C  L  E  D  F  F  Q  Q  R  P** *  
GTACAAGGCAATGGTATCCTGCCTGGAAGATTTTTTCCAGCAAAGACCATGA

Intron:1336bp

**LtF107**

***M  M  Q  R  G  A  V  L  L  G  V  V  A  F  L  A  L  W  P  Q***
ATGATGCAGAGAGGGGCCGTGCTGCTCGGGGTGGTCGCCTTCCTTGCCCTGTGGCCCCAG
***A  A  A*** K  V  Y  N  L  H  D  P  K  V  W  A  V  V  D  N  T  
GCCGCTGCCAAGGTGTACAATTTGCACGATCCTAAAGTGTGGGCCGTGGTCGACAATACA
R  R  **L  M  R  A  C  A  T  A  N  S  Y  M  D  N**             
CGGAGACTGATGCGCGCCTGTGCCACAGCCAACAGCTACATGGACAA*gtaagagagagag
agagagagagagagagagagagagagagagagagagagagagagagagagagagagaaag
agagagagactttgacactgatattttattgtcattacgaaagaggtcttttgacaaggg
gagggaggtaacagaacaataacatttccctcttcctttcctgcaaacaaatagtacact
ttcattcctagtgtgtatcaaatcaaaggttaacaaaaacaaataaattcgtaacatttc
cttatgtccccacgcacaggagtaataatacagtaaggtaaaaaaaaaaaaaaaaagaaa
gaaaagaaaaagaaacgaaaaaaaaaaatcgaaaagaaaaaaaactcttaagactgactc
gaccatccacccaatcaaatgataacattgtatcttactaatcacttccacatgcgatga
atgataacgttcattcatatatctcttataatgcattatcttaaatttcactatccttca
tctgtctaattttcaatgcttccgtgatgaatttcccaattgatttaattacatgttcat
catctgatgttaacacttcggcgacatattgtcttttcgctatatctaactgggaaaatg
aacaattcttgttttttgagagagagagagagagagagagagagagagagagagagagag
agagagagaggcgaaaagttgagtagtttcaacgtgaggtgactgtaggcggaaattgaa
acagattttaaaaaaaaatttaagttaactctgctggtctggtttacatggacaaagaaa
taaccagacaaaacatgacgcgtttcgacctcttaggtcttcttcaggcaagataacatt
caggacaatgaaagcgcaggaacgagcaatcaattgtcttctcgaattcggcgcaagacg
agcattgatggttcatgttaaccatgccaagtcatttacattttttttttaagaaaagaa
aaattcatcgcaagacacgctcttataaacctgctcacaaagcggaggaaactagggggt
ttgcaggaaacaaaggggtaaggaagttggggggaagaggagggaggggggcaaagaccg
acaaagggaaggggctataatattcaccccccccccccctctctctctctctgtaaacat
gtacacttgcgtgcgcgtgtgcatacctgcatgcgtgcatgcgtgtgtgtgtgtgtgtgt
gtgtgtgtgtgtgtgtgtgtgtgtgtgtgtgtgcgcggacgcgcacatccacttgtatgt*                             **P  W  S  Q  L  N  V  A  D  F  E***gtgtttgagtgtgtgacatacctttcag*CCCATGGTCTCAGTTGAACGTTGCGGACTTTG
  **E  R  S  R  Y  H  S  M  F  N  A  M  V  S  C  L  E  D  F  F**AAGAAAGGAGTCGATACCATTCAATGTTCAACGCCATGGTATCATGCCTGGAAGATTTTT
 **Q  Q  R  P***  
TCCAGCAAAGACCATGA

Intron:1301bp

**LtF108**

***M  M  Q  R  G  A  V  L  L  G  V  V  T  F  L  A  L  W  P  Q***ATGATGCAGAGAGGGGCCGTGCTGCTCGGGGTGGTCACCTTCCTTGCCCTGTGGCCCCAG
***V  A  A*** K  V  Y  D  L  Y  D  T  K  V  W  A  V  V  S  Y  S  
GTCGCTGCCAAGGTGTACGATTTGTACGATACTAAAGTGTGGGCCGTGGTCTCCTATTCA
K  G  **L  M  N  A  C  A  T  A  N  D  Y  M  D  D**             
AAGGGACTGATGAACGCCTGTGCCACAGCCAATGACTACATGGACGA*gtaagagagagag
agagagagagagagagagagagagagagagagagagactttgacactgatattttattgt
cattacgaaagaggtcttttgacaaggggagggaggtaacagaacaataacatttccctc
ttcctttactgcaaacaaatggtacactttcattcctagtgtgtatcaaatcaaaggtta
acaaaaacaaatacattcgtaacatttccttatgtccccacgcacaggagtaatacagta
aggtgaaaaaaaaaaaaagaaaagaaaaagaaacgaaaaaaaaatcgaaaaaaaaccctt
aagactgactcgaccatccgcccaatcaaatgatgacattgtatcttactaatcacttcc
acatgcgatgagtgataatgttaattcatatatctcttataatgtattatcttaaatttc
actatccttcatctgtcttattttcaatgcttccgtgatgaatttcgcaattgatttaac
tacatgttcatcatctgatgataacacttcggcgacattttgtcttttcgttatatctaa
ctggaaaaatgaacaattcttgtttgttttttttttttgttttttgttgttttttttttt
ttttttttttttgagagagagagaaaagagagagagagagagagagagagagagaggcga
aaagttgaatagtttcaacgtgaggggactgtaggcggaaattgaaacaaatttttaaag
taattttaaggtaactccaccggtttacatggacaaagaaataaccagacaaaacatgac
gcgtttcgacctcttaggtcttcttcaggcaagataacattcaggacaatgaaaacgcag
gaacaagcaatcaattgtcttcttgaattcggcgcaagatgagcattgatggttcatgtt
aaccatgccaagtcatttacttttttttttaagaaaagaaaagttttatcacaagacacg
ctcttataaacctgcaggacaaaggggaggaaactagggggtttgcggggaacaaagggg
tgaggaagttggggggaagaggagggaggggggcaaagaccgacaaagggaaggggctat
aatattcaccctctctctctctctctctctctctctctctctctctctctctctctctct
ctctctgtgtaaacatgtacacttgcgtgaacgtgtgcatacctgcatacgtgcatgagt
gtgtgtgtgtatgtgtgtgtgtgtgtgtgtgtgtgtgtgcgtgtgcgtgtgtgtgtgtgt
gtgtgtgtgtgtgcacacgcgcacatccgcttgtatgtgtgtttgtgtgtgtgacatatc*       **P  W  S  V  V  D  V  G  D  F  E  E  R  S  L  Y  N  S**
*tttcag*CCCATGGTCTGTGGTGGATGTTGGGGACTTTGAAGAAAGGAGTCTATACAATTC
 **L  Y  K  A  M  V  S  C  L  E  D  F  F  Q  Q  R  P***  
ATTGTACAAGGCAATGGTATCCTGCCTGGAAGATTTTTTCCAGCAAAGACCATGA

Intron:1339bp
